# Mapping neurodegeneration with diffusion MRI: biomarkers, mechanisms, and clinical translation

**DOI:** 10.3389/fneur.2026.1757762

**Published:** 2026-03-13

**Authors:** Francesca Bozzetti, Antonino Errante, Daniele Corbo, Roberto Gasparotti, Marco Salvi, Fulvio Lauretani, Nicola Sverzellati

**Affiliations:** 1 Neuroradiology Unit, Diagnostic Department, University Hospital of Parma, Parma, Italy; 2 Department of Medicine and Surgery, University of Parma, Parma, Italy; 3 Centro Cardinal Ferrari - KOS, Parma, Italy; 4 Department of Medical and Surgical Specialties, Radiological Sciences and Public Health, University of Brescia, Brescia, Italy; 5 University Hospital of Parma, Medicine and Geriatric-Rehabilitation Department, Clinic Geriatric Unit and Cognitive and Motor Center, Parma, Italy; 6 Unit of Radiological Sciences, Diagnostic Department, University Hospital of Parma, Parma, Italy

**Keywords:** diffusion MRI, free-water imaging, glymphatic system, microstructural imaging, neurite density, neurodegeneration, perivascular spaces (PVS)

## Abstract

Neurodegenerative diseases share convergent mechanisms involving microstructural degeneration, neuroinflammation, vascular dysfunction, and impaired brain fluid homeostasis. The neurovascular unit (NVU) represents a critical interface where these processes interact, integrating neuronal, glial, vascular, and perivascular components that regulate metabolism, immune surveillance, and waste clearance. This review examines advanced diffusion MRI as a noninvasive framework to investigate NVU-related pathology, with a specific focus on tissue microstructure, water dynamics, and perivascular spaces (PVS). We summarize diffusion MRI techniques ranging from conventional diffusion tensor imaging to multi-compartment and biophysical models that probe neurite architecture, extracellular free water, and perivascular transport. Across aging and major neurodegenerative disorders, diffusion-derived markers consistently reveal microstructural disorganization, extracellular fluid expansion, PVS enlargement, and glymphatic dysfunction. These alterations reflect coupled tissue–fluid pathology rather than isolated cellular damage. While advanced diffusion approaches provide increased sensitivity to early and subtle changes, they are influenced by acquisition quality, model assumptions, physiological confounders, and limited histopathological validation. Importantly, diffusion MRI metrics should be interpreted as complementary biomarkers that enhance, but do not replace, established diagnostic criteria and molecular biomarkers for specific neurodegenerative diseases. When integrated within multimodal and longitudinal frameworks, diffusion MRI offers valuable insights into NVU dysfunction, supporting early disease stratification, progression monitoring, and mechanistic understanding of neurodegeneration.

## Introduction

Neurodegenerative diseases represent a major public health challenge due to their high prevalence, profound impact on quality of life, and limited therapeutic options. Early and accurate biomarkers are essential to enable timely diagnosis, disease stratification, and monitoring of progression, particularly during preclinical and prodromal stages.

Across neurodegenerative disorders, convergent pathological mechanisms involve microstructural degeneration, neurovascular unit (NVU) dysfunction, neuroinflammation, and impaired brain fluid homeostasis. Diffusion-based MRI provides a unique, noninvasive window into these processes by probing tissue microstructure and water dynamics at spatial scales inaccessible to conventional MRI.

At the macrostructural level, gray matter (GM) and white matter (WM) differ markedly in cellular composition and microarchitecture, critically shaping diffusion metrics and necessitating tissue-specific interpretation of microstructural biomarkers (1).

This review presents an integrated framework describing how diffusion MRI captures the microstructural and fluid-dynamic substrates of neurodegeneration. We first outline diffusion MRI techniques used to assess tissue microstructure, extracellular free water, and perivascular spaces (PVS). We then summarize diffusion-derived alterations across aging and major neurodegenerative diseases. Finally, we integrate these findings to identify diffusion-based biomarkers with potential clinical relevance for early diagnosis, stratification, and longitudinal monitoring.

## Neurovascular unit

The NVU is a highly integrated cellular network that regulates cerebral blood flow in accordance with neuronal and glial metabolic demands. It also orchestrates the bidirectional exchange of solutes and immune cells between the peripheral circulation and the brain parenchyma through the blood–brain barrier (BBB), while exerting critical control over neuroinflammatory responses via complex intercellular signaling pathways (2).

The activity of the NVU is further shaped by circadian regulation. Central pacemaker structures impose oscillatory control over vascular tone, BBB permeability, metabolic coordination, and glial–activity.

The NVU comprises endothelial cells, pericytes, vascular smooth muscle cells, astrocytic end-feet, neurons, microglia, and extracellular matrix components.

In addition to this systemic rhythm, local cellular oscillators within endothelial, neuronal, and glial compartments contribute to the fine modulation of neurovascular dynamics. Disruption of these circadian mechanisms increases susceptibility to neurological and psychiatric disorders (3).

**Figure 1:**
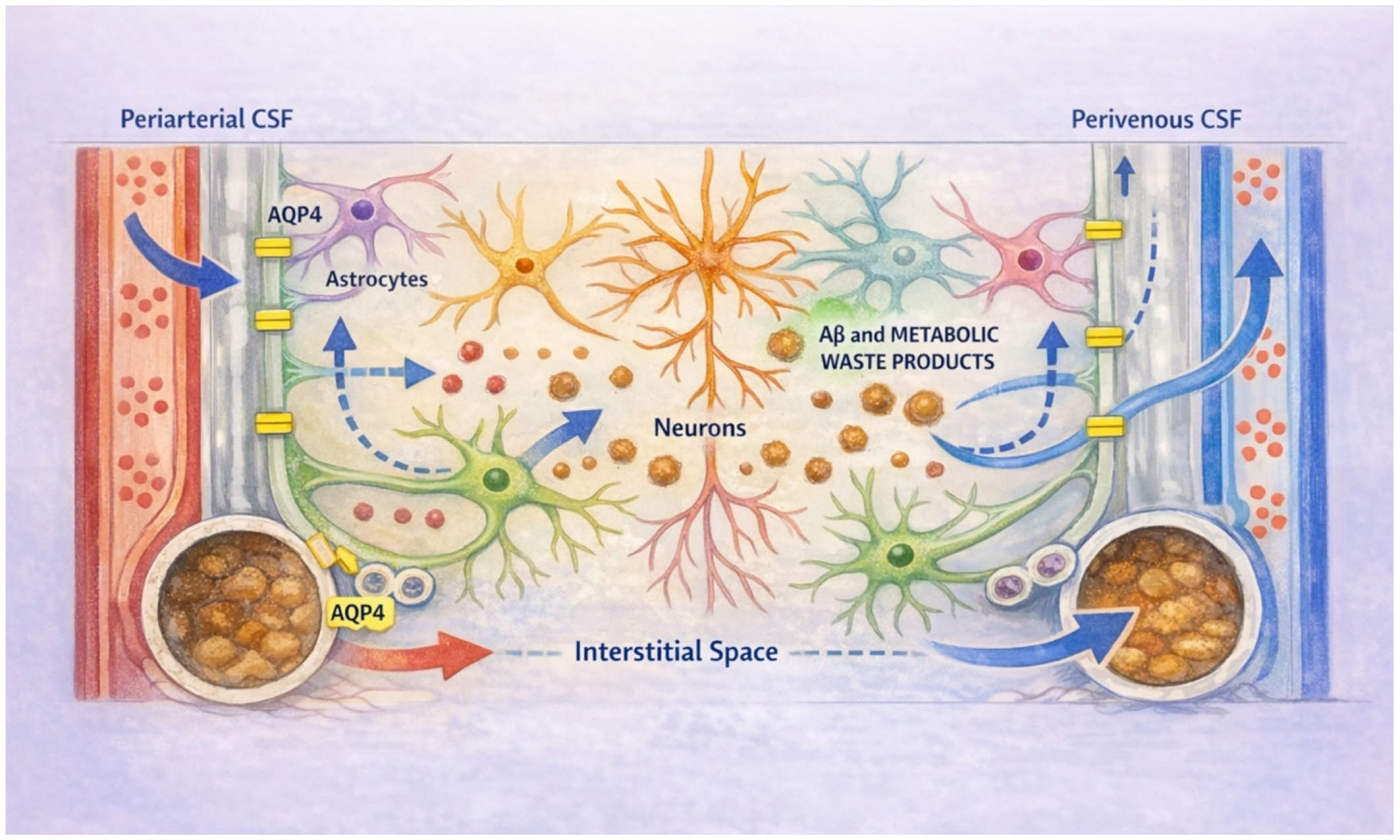
CSF enters the brain along periarterial spaces, facilitated by aquaporin-4 (AQP4) water channels expressed on astrocytic end-feet. CSF mixes with interstitial fluid within the brain parenchyma, promoting the clearance of soluble amyloid-*β* (Aβ) and other metabolic waste products. This bulk flow–driven mixture exits along perivenous spaces and is ultimately drained into lymphatic vessels for elimination.

Aging introduces profound structural and functional alterations in the NVU, including arterial, venous, and lymphatic remodeling. These changes contribute to reduced oxygen–glucose delivery, impaired endothelial transport, diminished clearance of neurotoxic proteins, compromised immune surveillance, and attenuated neurotrophic signaling. Collectively, these processes enhance the vulnerability of the aging brain to neurodegenerative pathology (4). Microstructural interpretation is supported by advanced diffusion models (Figure 1).

## Diffusion techniques

### Diffusion-weighted imaging (DWI)

Diffusion-weighted imaging (DWI) is a foundational MRI technique that provides high sensitivity to early microstructural tissue alterations by probing water mobility at the microscopic scale (5). In clinical practice, DWI serves as a rapid screening tool for detecting acute pathological changes—most notably cytotoxic edema—often before abnormalities become apparent on conventional structural imaging (6, 7).

In neurodegenerative and inflammatory conditions, DWI abnormalities reflect composite changes in tissue organization rather than a single biological process. Consequently, diffusion signal alterations should be interpreted as markers of altered tissue state rather than direct surrogates of specific cellular mechanisms (8, 9). For this reason, DWI is best viewed as an entry-level diffusion technique whose findings require contextualization using more specific diffusion models.

The physical determinants of diffusion signal behavior, quantitative parameters, and principal clinical applications of DWI are summarized in Table 1, which provides the technical framework supporting routine and research-oriented interpretation.

**Table 1:** Key concepts, interpretation, and limitations of diffusion-weighted imaging (DWI).

Topic	Key concepts and interpretation	Main limitations	Supporting references
Historical background and principle	DWI encodes the random displacement of water molecules using motion-sensitive gradient pulses; signal attenuation reflects tissue diffusivity and enables sensitive detection of early microstructural alterations.	Provides indirect measures of tissue microstructure; diffusion signal reflects multiple overlapping physical and biological processes rather than a single cellular mechanism.	(5)
Primary quantitative metric (ADC)	The apparent diffusion coefficient (ADC) reflects overall water mobility within a voxel and integrates Gaussian and non-Gaussian diffusion components, IVIM-related pseudodiffusion, and b-value dependence.	Limited biological specificity; ADC changes cannot distinguish between demyelination, edema, inflammation, or neuronal loss without complementary models.	(6, 8, 9)
Biophysical determinants	DWI and ADC are influenced by membrane permeability, macromolecular interactions, extracellular matrix composition, and microvascular flow.	Susceptible to confounding effects from perfusion, extracellular free water, and partial-volume contamination, particularly near CSF spaces.	(54)
Clinical utility	Fast, robust, and widely available; essential for acute stroke imaging and routinely applied in neuro-oncology, infection, inflammation, and neurodegenerative disease assessment.	Primarily a screening tool; limited sensitivity to subtle or chronic microstructural changes compared with advanced diffusion models.	(7, 11, 52, 53)
Technical considerations	Compatible with standard single-shell acquisitions and short scan times.	Sensitive to acquisition parameters, motion, susceptibility artifacts, and scanner variability; reduced reliability in gray matter and regions with low anisotropy.	(8, 9)

### Diffusion MRI phenomenological and biophysical models

Diffusion MRI has progressively emerged as a central tool for the *in vivo* investigation of brain microstructure due to its unique ability to probe microscopic water displacement within tissues. Unlike conventional MRI, which is limited by millimeter-scale spatial resolution, diffusion techniques enable indirect characterization of cellular and subcellular architecture at the micrometer scale (6, 8).

The sensitivity of diffusion MRI to different tissue compartments is governed by the degree of diffusion weighting, modulated by the b-value. Low b-values primarily capture fast-moving or extracellular water components, including perfusion-related signal contributions, whereas higher b-values emphasize restricted diffusion within axons and dendrites (10). The introduction of multi-shell acquisition strategies has represented a major methodological advance, enabling the development of increasingly sophisticated diffusion models with improved microstructural specificity (7, 9, 11, 12).

Phenomenological models such as diffusion tensor imaging (DTI) describe diffusion using simplified mathematical representations, assuming Gaussian diffusion behavior. DTI provides robust and reproducible metrics, including fractional anisotropy (FA) and mean diffusivity (MD), which are highly sensitive to macro- and microstructural tissue alterations but lack biological specificity. Extensions such as diffusion kurtosis imaging (DKI) incorporate non-Gaussian diffusion effects, increasing sensitivity to microstructural complexity in both white and gray matter (8).

Biophysical models aim to overcome these limitations by explicitly modeling tissue compartments. Techniques such as NODDI, CHARMED, SMT, AxCaliber, ActiveAx, WMTI, and DBSI attempt to disentangle intra-axonal, extra-axonal, and isotropic water components, providing more biologically interpretable metrics related to neurite density, orientation dispersion, axonal diameter, and extracellular water content (9, 11). However, these models rely on strong assumptions regarding tissue geometry, diffusivity constraints, and water exchange, and they typically require high-quality multi-shell acquisitions with increased scan time.

Table 2 provides a summary of the principal models, including both phenomenological and biophysical approaches.

**Table 2:** Advanced diffusion MRI models: characteristics, strengths, limitations, acquisition requirements, and post-processing.

Technique	Main characteristics	Typical directions/shells	Min. SNR (approx.)	Typical post-processing steps	Strengths	Limitations	Specific applications
DTI	Tensor-based Gaussian diffusion model; ≥1 non-zero *b*-value (≤1,000–1,500 s/mm^2^).	≥6–12 directions, 1 shell	≥15–20	Eddy-current & motion correction; brain masking; tensor fitting; FA/MD/AD/RD maps	Simple, robust, low acquisition burden	Low biological specificity; crossing fibers unresolved	Global WM integrity; gross microstructural damage
DKI	Extension of DTI capturing non-Gaussian diffusion; ≥2 *b*-values (≤2,500–3,000 s/mm^2^).	≥30 directions, ≥2 shells	≥20–25	DTI preprocessing; kurtosis fitting; MK/AK/RK maps	Higher sensitivity to microstructural complexity	Noise-sensitive; higher scan time	WM and GM microstructural heterogeneity
CHARMED	Two-compartment intra−/extra-axonal model; fixed axon diameter; multi-shell.	≥60–90 directions, ≥3 shells	≥25–30	Denoising; distortion correction; multi-compartment model fitting	Improved axonal specificity	Strong assumptions; high data demand	Axonal density and extra-axonal diffusion
AxCaliber	Restricted + hindered compartments; gamma-distributed axon diameters; multi–diffusion-time.	≥90–120 directions, multi-shell & multi-time	≥30	Advanced motion correction; model fitting across diffusion times	Estimates axon diameter distribution	Very high acquisition burden; research-only	Detailed axonal morphometry
ActiveAx	Simplified axon diameter + density model; fixed diffusivities.	≥60 directions, ≥2 shells	≥25	Standard preprocessing; constrained model fitting	Lower complexity than AxCaliber	Oversimplified assumptions	Axonal metrics in coherent tracts
WMTI	Two-compartment model for single-fiber regions; DKI-based.	≥30 directions, ≥2 shells	≥20–25	DKI preprocessing; WMTI parameter estimation	Uses standard DKI data	Limited to coherent fibers	Axonal water fraction in CC
NODDI	Three-compartment neurite model; fixed diffusivities; Watson/Bingham ODF.	≥30–60 directions, 2 shells (~1000/3000)	≥20–25	Motion/distortion correction; NODDI fitting; NDI/ODI/fISO maps	Widely used; WM and GM applicability	Sensitive to inflammation/edema	Neurite density and orientation dispersion
MC-SMT	Two-compartment intra−/extra-neurite model; equal diffusivities.	≥30–60 directions, 2 shells	≥20–25	Standard preprocessing; SMT fitting	Reduced orientation bias	Strong constraints; less intuitive	Orientation-independent microstructure
DBSI	Multi-compartment anisotropic + isotropic model; no exchange.	≥90–100 directions, multi-shell	≥30	Extensive preprocessing; voxel-wise multi-compartment fitting	Separates axonal injury, edema, cellularity	Very high complexity; limited standardization	Inflammatory/demyelinating pathology

The reported acquisition requirements—including the number of diffusion directions, signal-to-noise ratio (SNR), and post-processing steps—should be interpreted as indicative reference values, as they depend on magnetic field strength, voxel size, diffusion weighting, and population-specific factors (e.g., adult versus fetal imaging). While standardized preprocessing pipelines typically include denoising, motion and distortion correction, and model-specific fitting, acquisition feasibility and robustness vary substantially across diffusion models. It should be noted that many of the cited studies do not fully meet these standards, particularly with respect to acquisition harmonization, minimum SNR requirements, and completeness of preprocessing pipelines.

From a clinical translation perspective, DTI currently demonstrates high clinical readiness, owing to its robustness, low acquisition demands, and widespread availability. DKI and NODDI exhibit moderate clinical readiness, offering improved microstructural sensitivity at the cost of increased acquisition time and increased sensitivity to noise and model assumptions. In contrast, higher-order biophysical models such as CHARMED, AxCaliber, ActiveAx, MC-SMT, and DBSI remain largely experimental, given their stringent acquisition requirements, complex modeling, and limited standardization, which currently restrict their routine clinical use.

Beyond feasibility, the interpretation of diffusion-derived metrics remains inherently challenging. These parameters are influenced not only by disease-related microstructural alterations but also by aging, physiological state, acquisition protocol, and model-specific assumptions. For instance, increased radial diffusivity may reflect demyelination, extracellular edema, or generalized tissue rarefaction, whereas elevated mean diffusivity may arise from neuronal loss, vasogenic processes, or changes in extracellular water content (8, 10).

Additional limitations include partial-volume effects, signal-to-noise constraints, and substantial protocol heterogeneity across scanners and studies. A critical source of variability lies in the distinct microstructural organization of white matter (WM) and gray matter (GM), which differ markedly in cellular composition, anisotropy, fiber orientation dispersion, and dendritic complexity. Consequently, diffusion models optimized for WM may be suboptimal—or potentially misleading—when applied to cortical or deep GM regions, where microstructural heterogeneity is substantially greater (1).

Multidimensional diffusion approaches that exploit variations in diffusion time, b-tensor shape, and encoding schemes have demonstrated advantages in separating isotropic from anisotropic diffusion components and improving microstructural specificity, particularly in GM (1). However, the reliability of advanced diffusion models remains strongly dependent on acquisition quality. Optimal performance typically requires multi-shell data spanning approximately 1,000–3,000 s/mm^2^, sufficient angular resolution, appropriate diffusion-time optimization, and rigorous control of cerebrospinal fluid partial-volume contamination, especially in periventricular regions (13–16).

In the absence of these precautions, model estimates may become unstable, poorly reproducible, or biologically implausible. Intrinsic limitations further complicate interpretation, including sensitivity to noise and motion, oversimplified microstructural assumptions, inaccurate modeling of crossing fibers, and potential overestimation of neurite density in inflammatory contexts or when extracellular signal is reduced (11, 17).

Overall, these considerations reinforce that clinical applicability reflects a trade-off between biological specificity and practical feasibility, underscoring the need to balance methodological sophistication with robustness, reproducibility, and interpretative caution in both research and clinical settings.

### Diffusion microstructure imaging

Diffusion microstructure imaging (DMI) is an advanced diffusion MRI technique that uses a Bayesian framework to estimate tissue microstructure properties from diffusion-weighted imaging data. It models three compartments—free water (V-CSF), intra-axonal (V-intra), and extra-axonal (V-extra)—and their corresponding diffusivities, enabling separation of macroscopic fiber geometry from microstructural features such as axon density and diameter, even in the presence of crossing fibers. Compared with conventional approaches (DTI, DKI, NODDI), DMI provides superior microstructural specificity using standard acquisition schemes.

However, partial-volume effects, particularly in atrophic regions, and the need for robust histopathological validation remain important limitations (18).

### Tractography

Connectomes, typically represented as matrices or graphs, describe the topological organization and efficiency of connections between brain regions. Graph theory enables the derivation of quantitative metrics from structural and functional connectomes that are sensitive to variations in brain connectivity related to development, aging, and disease. These metrics can be assessed at global and local levels, allowing identification of regional connectivity hubs and subnetworks that contribute to the brain’s structural and functional architecture (19).

Within this framework, diffusion-based tractography enables *in vivo* reconstruction of large-scale white matter connectivity but remains constrained by technical and biological limitations. In regions of fiber crossing or complex architecture, ambiguous diffusion signals increase the risk of false-positive streamlines, whereas long-range, thin, or highly curved projections may be underrepresented due to spatial resolution limits, leading to false negatives (20). Additional challenges at the white matter–gray matter interface, including partial volume effects and low anisotropy, further compromise cortical connectivity mapping.

Even probabilistic tractography may misrepresent fine-scale connectivity under conditions of low signal-to-noise ratio or complex diffusion profiles (21). Despite these limitations, tractography-based structural connectomics has contributed valuable insights into network-level alterations in neurodegenerative diseases. When integrated with biophysical diffusion models—such as NODDI, SMT, and free-water imaging—and with perivascular biomarkers including ALPS and perivascular space metrics, tractography supports a multimodal framework for investigating microstructural damage, glymphatic dysfunction, and network-based propagation of pathology.

However, specific false-positive and false-negative connections were consistently identified across different tractography methods. Although the challenge dataset does not capture the full complexity of the human brain, it provides unique data with known macrostructural and microstructural ground-truth properties, enabling a rigorous evaluation of connectivity estimation approaches (22).

## Production to clearance: an integrated fluid-exchange system

**Figure 2:**
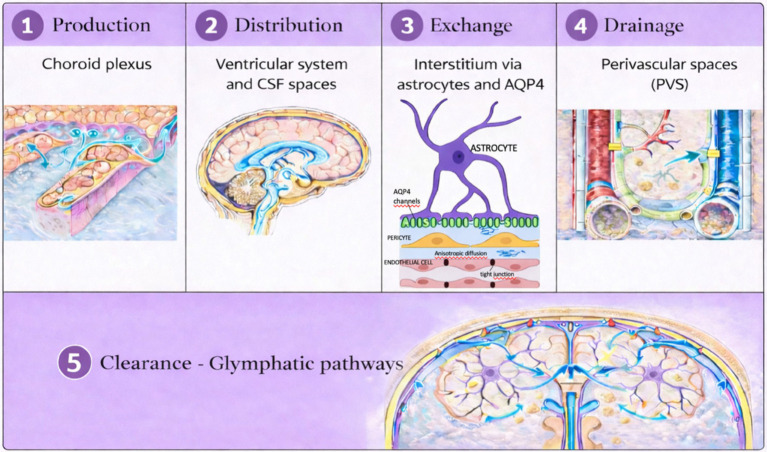
Overview of cerebrospinal fluid (CSF) circulation and clearance. CSF is produced by the choroid plexus, distributed through the ventricular system and CSF spaces, exchanges with interstitial fluid via astrocytic AQP4 channels, drains along perivascular spaces, and is ultimately cleared through the glymphatic system.

Diffusion-based MRI techniques allow a unified characterization of the entire cerebral fluid circulation (Figure 2):

1. Production — Choroid plexus.
2. Distribution — Ventricular system and CSF spaces.
3. Exchange — Interstitium via astrocytes and AQP4.
4. Drainage — Perivascular spaces (PVS).
5. Clearance — Glymphatic pathways.

### Choroid plexus in neurodegeneration

The choroid plexus (CP) plays a central role in CSF production, immune surveillance, and regulation of the blood–CSF barrier, thereby exerting a critical influence on glymphatic function and overall brain homeostasis. Its intraventricular location places it at a strategic interface between CSF circulation and interstitial fluid (ISF) exchange, making it particularly relevant for metabolic waste clearance.

Age-related CP changes have been consistently reported, with progressive volumetric enlargement accompanied by increased mean diffusivity, reflecting microstructural damage and increased water mobility (23). Despite this enlargement, CP functional capacity declines with aging, as evidenced by reduced CSF secretion, suggesting a dissociation between structural hypertrophy and physiological efficiency.

In Alzheimer’s disease (AD), CP alterations appear to play a more direct pathological role. Choroid plexus free-water fraction (CP-FWf) has been introduced as a diffusion-based biomarker of cerebral water circulation and glymphatic efficiency (24). Elevated CP-FWf is associated with amyloid-*β* deposition, reduced DTI-ALPS values, brain atrophy, tau burden, synaptic loss, and cognitive decline, supporting its role as an early marker of impaired clearance mechanisms. Longitudinal data further indicate that CP-FWf increases more rapidly in the presence of amyloid pathology than other imaging markers, and correlates with white matter hyperintensity burden, linking glymphatic dysfunction to small-vessel–related tissue damage (24) validated by PET.

### Diffusion MRI assessment of CSF and free-water dynamics

#### CSF dynamics

Diffusion MRI is currently the most versatile noninvasive technique for investigating CSF dynamics, owing to its sensitivity to incoherent water motion across the brain. CSF is characterized by low viscosity, low protein content, and long T2 relaxation time, properties that can be exploited—particularly with longer echo times—to suppress tissue and blood signal and enhance sensitivity to fluid motion (25).

Several diffusion-based approaches have been applied to CSF assessment. Qualitative low–b-value DWI (<1,000 s/mm^2^) is sensitive to bulk fluid motion but remains influenced by cardiac pulsation and subjective interpretation. Conventional DTI acquired at low b-values has also been used, although with limited specificity and prolonged acquisition times. The DTI-ALPS method was specifically introduced to assess perivenous CSF outflow by exploiting directional diffusivity asymmetries in deep white matter.

More advanced multi-compartment diffusion models address these limitations by separating fluid-related signal contributions from tissue microstructure. Multi–b-value DWI enables explicit modeling of free-water diffusion, allowing differentiation between extracellular fluid expansion and true microstructural alterations. IVIM imaging is sensitive to increases in interstitial fluid, particularly during sleep, although higher b-values may introduce kurtosis-related confounds. NODDI provides complementary information by estimating extracellular free-water content in conditions characterized by enlarged PVS and increased interstitial fluid. Spectral diffusion analysis further extends this framework by identifying a distinct diffusion compartment encompassing both interstitial and perivascular fluid.

In diffusion MRI, CSF within ventricles, subarachnoid spaces, and edema is treated as free water. Signal contamination from CSF introduces partial-volume effects that bias diffusion metrics and tractography, particularly in neurodegenerative diseases where subtle microstructural changes are of interest. To mitigate this issue, the Free Water Elimination (FWE) model proposed by Pasternak et al. was developed to improve the specificity of diffusion metrics to tissue microstructure (26).

#### Free water imaging (FWI)

Free Water Imaging (FWI) is a diffusion MRI approach designed to separate extracellular free water from tissue-specific diffusion signals using a bi-compartment model. In this framework, the diffusion signal is modeled as the combination of an isotropic free-water compartment—representing cerebrospinal and interstitial fluid—and a tissue compartment reflecting white-matter microstructure. This separation reduces CSF-related partial-volume effects and increases the biological specificity of diffusion measurements, particularly in conditions characterized by edema or altered fluid dynamics.

The principal FWI-derived metric is the free-water fraction (FW), which quantifies the relative contribution of extracellular freely diffusing water within each voxel. In addition, free water–corrected diffusion tensor metrics—including FA_t, MD_t, AD_t, and RD_t—can be derived, enabling a more accurate assessment of tissue microstructure independent of extracellular contamination.

Traditional diffusion indices such as mean diffusivity and fractional anisotropy reflect composite effects of multiple processes, including edema, demyelination, axonal degeneration, and CSF partial-volume contamination. As highlighted by Martinez-Heras et al. (11), Jelescu et al. (8), and Le Bihan (10), these metrics lack specificity for isolating nonspecific extracellular water and may therefore confound interpretation in neuroinflammatory and neurodegenerative conditions.

Methodological and physiological advantages of FWI have been further clarified by (27). While MD reflects average diffusivity across both intra- and extracellular compartments and RD is primarily influenced by myelin integrity, neither metric can independently quantify extracellular water. FWI overcomes this limitation by explicitly modeling the free-water compartment, allowing more precise characterization of pathological processes associated with extracellular space expansion, neuronal loss, blood–brain barrier disruption, and inflammatory activity.

Diffusion-derived metrics sampled along perivascular trajectories provide mechanistically complementary information on interstitial and perivascular fluid dynamics. Regional variations in diffusion anisotropy and radial diffusivity within deep white matter reflect alterations in fluid mobility and extracellular organization that parallel microstructural disruption, as demonstrated in neurodegenerative and inflammatory conditions (28).

Increases in free-water fraction represent extracellular space expansion driven by vasogenic edema, neuroinflammation, blood–brain barrier dysfunction, and impaired interstitial–glymphatic clearance (29). By explicitly modeling and removing the isotropic extracellular compartment, free water imaging (FWI) disentangles fluid-related signal contributions from tissue-specific diffusion properties, thereby improving the biological specificity of diffusion metrics.

Within this framework, FWI captures a convergent pathological substrate shared across neurodegenerative, neuroinflammatory, and cerebrovascular disorders, where extracellular fluid accumulation reflects both structural tissue injury and failure of perivascular and glymphatic clearance mechanisms. When integrated with conventional diffusion tensor metrics and advanced microstructural models, FWI enables a more comprehensive characterization of the coupled tissue–fluid pathology underlying disease progression and clinical decline (Table 3) (28, 29).

**Table 3:** Free-Water Imaging (FWI): strengths and limitations.

Strengths	Limitations
Separates extracellular free water from tissue-specific diffusion, improving biological specificity compared with conventional DTI.	Free-water increase is indirect and non–disease-specific, reflecting a combination of neurodegeneration, inflammation, edema, BBB dysfunction, and impaired clearance.
Highly sensitive to early microstructural alterations, often preceding macroscopic atrophy and clinical symptom onset.	Based on simplified bi-compartment models that may not fully capture tissue and cellular complexity.
Reduces CSF-related partial-volume effects, particularly in cortical and periventricular regions.	Sensitive to acquisition quality, noise, and motion, which may affect estimation stability and reproducibility.
Shows strong associations with cognitive decline and disease severity across neurodegenerative and neuroinflammatory disorders (AD, PD, MS, NMOSD).	Limited direct histopathological validation in human studies.
Applicable to both gray and white matter and scalable to large clinical and multicenter datasets.	Interpretation requires integration with multimodal imaging, clinical, and biological biomarkers.

#### Diffusion-based markers of fluid homeostasis

Kurtosis metrics capture deviations from Gaussian diffusion and reflect microenvironmental complexity. Reductions in cortical mean and radial kurtosis have been linked to decreased diffusion restriction and increased fluid mobility within reorganized microenvironments (30) NODDI further refines this assessment by decomposing diffusion signal into intracellular and extracellular components, particularly in periventricular and deep gray matter regions where fluid-related effects are prominent.

### Astrocytes, AQP4, and interstitial–perivascular exchange

Astrocytes regulate water transport between interstitial and perivascular compartments through polarized expression of aquaporin-4 (AQP4) channels at their endfeet. Proper AQP4 localization is essential for efficient interstitial fluid movement and perivascular exchange. Disruption of AQP4 expression or polarization impairs interstitial flow, leading to extracellular fluid accumulation and progressive microstructural disorganization. These alterations are detectable with diffusion MRI, providing a mechanistic link between cellular dysfunction and imaging-based markers of fluid dysregulation (31).

### Perivascular spaces (PVS) and blood–brain barrier interplay

Perivascular spaces (PVS) are anatomical channels surrounding penetrating arterioles, capillaries, and small venules, forming a critical interface between cerebrospinal fluid (CSF) and the interstitial compartment of the brain. Under physiological conditions, PVS are narrow and typically not visible on conventional MRI. When enlarged, however, they emerge as sensitive imaging markers of vascular pathology, neuroinflammation, and glymphatic dysfunction (32).

Functionally, PVS facilitate the exchange of solutes between CSF and interstitial fluid (ISF), supporting metabolic waste clearance and contributing to brain fluid homeostasis. Their efficiency depends on the coordinated integrity of the neurovascular unit, including vascular pulsatility, blood–brain barrier (BBB) function, and the polarized expression of aquaporin-4 (AQP4) channels on astrocytic endfeet. Enlargement of PVS is increasingly recognized as a structural signature of impaired perivascular transport, reduced glymphatic flow, and compromised clearance capacity.

BBB disruption and PVS enlargement represent interdependent pathological processes across cerebral small vessel disease and neurodegenerative disorders. Increased BBB permeability allows serum-derived neurotoxic molecules to infiltrate the brain parenchyma, promoting vasogenic edema and neuroinflammatory responses. In parallel, PVS dilation reflects stagnation of interstitial fluid and accumulation of metabolic waste products, indicating impaired perivascular drainage.

The BBB is responsible for approximately 60–85% of amyloid-*β* (Aβ) clearance from the brain, with the remaining fraction mediated via perivascular and glymphatic pathways. Consequently, BBB failure accelerates Aβ retention within perivascular compartments, promoting further PVS enlargement. Accumulated Aβ, in turn, disrupts AQP4 polarization on astrocytic endfeet—a key determinant of glymphatic inflow—thereby further impairing perivascular transport efficiency. This establishes a self-reinforcing cycle in which BBB dysfunction, impaired glymphatic clearance, and progressive PVS enlargement mutually exacerbate one another, leading to sustained perivascular dysfunction and reduced metabolic clearance (Table 4) (33).

**Table 4:** Strengths and Limitations of Perivascular Spaces (PVS) as Imaging Biomarkers.

Strengths	Limitations
Reflect key mechanisms of glymphatic function and CSF–ISF exchange	Limited spatial resolution on conventional MRI, especially for small PVS
Sensitive to early vascular and glymphatic dysfunction (e.g., MCI)	Difficulty distinguishing PVS from small lacunes or WMH in some regions
Noninvasive and detectable on routine MRI sequences	High inter-rater variability with visual rating scales
Correlate with free-water metrics, ALPS index, BBB leakage, and astrocytic markers (e.g., GFAP)	Lack of standardized segmentation and quantification methods
Provide insight into neurovascular unit (NVU) dysfunction	Regional heterogeneity (centrum semiovale vs. basal ganglia) complicates interpretation
Relevant across multiple disorders (AD, PD, MS, NMOSD/MOGAD)	PVS enlargement is not disease-specific
Can be integrated with diffusion MRI for microstructural and fluid-sensitive assessment	Influenced by age, vascular risk factors, and imaging protocol
Potential surrogate marker of impaired waste clearance	Causality between PVS enlargement and neurodegeneration remains unclear

### The Glymphatic system: physiological framework and diffusion MRI assessment

#### The glymphatic system

The glymphatic system is a brain-wide clearance pathway responsible for the exchange and removal of metabolic waste products from the central nervous system. Its function can be conceptually divided into three sequential phases involving cerebrospinal fluid (CSF) production, parenchymal exchange, and interstitial fluid (ISF) clearance (34).

Disruption of any component of this system—CSF production, perivascular influx, astrocytic AQP4 function, or perivenous drainage—results in impaired glymphatic clearance and contributes to fluid stagnation, metabolic stress, and neurodegeneration.

From an imaging perspective, glymphatic function can be indirectly assessed using diffusion MRI–based approaches. The DTI-ALPS method provides a functional marker of perivascular fluid transport along medullary veins. Diffusion kurtosis imaging (DKI) captures changes in microstructural complexity associated with altered interstitial environments. Analysis of perivascular spaces (PVS) offers a structural marker of impaired fluid drainage, while free-water imaging quantifies extracellular fluid expansion reflecting disrupted CSF–ISF exchange. Together, these techniques enable a multiparametric, noninvasive characterization of glymphatic system integrity *in vivo*.

This mechanistic model integrates noradrenergic neuromodulation, vascular vasomotion, and glymphatic transport into a unified multiscale framework, highlighting how coordinated LC-driven oscillatory activity supports perivascular fluid dynamics and brain waste clearance, particularly during sleep (Table 5) (35).

**Table 5:** Mechanistic model of the noradrenergic vasomotion–glymphatic axis.

Level	Component	Mechanism	Functional consequences
Central (master oscillator)	Locus coeruleus (LC)	Tonic and infralow-frequency bursting of noradrenergic neurons generates rhythmic volume transmission of norepinephrine (0.01–0.1 Hz), entraining vascular and astrocytic activity and synchronizing vascular tone and perivascular conductance	Coordinates coherent vasomotion; degeneration, sleep loss, or inflammation disrupts rhythmic drive and abolishes coordinated vasomotion
Vascular effector	Vascular smooth muscle cells (VSMCs)	α1-adrenergic Gq/PLC/IP3–mediated Ca^2+^ cycling generates low-frequency contraction–relaxation cycles, modulated by β2-adrenergic signaling	Produces sustained convective pressure gradients driving perivascular fluid transport; vascular stiffening dampens oscillations and reduces hydraulic efficiency
Microvascular	Pericytes	α1-adrenergic–dependent actin–myosin contractility fine-tunes capillary resistance and propagates vasomotor waves	Maintains phase-coherent microvascular perfusion; pericyte loss leads to heterogeneous constriction and impaired clearance
Glial interface	Astrocytic end-feet	Noradrenergic Ca^2+^ waves regulate aquaporin-4 (AQP4) localization, cytoskeletal structure, end-foot volume, and perivascular resistance	Couples CSF influx with interstitial fluid exchange; AQP4 depolarization increases resistance and promotes glymphatic failure
Conduit	Perivascular spaces (PVS)	Act as compliant channels integrating cardiac, respiratory, and vasomotor oscillations, with slow vasomotion as the dominant sustained driving force	Enables efficient solute transport from periarterial to perivenous compartments; reduced compliance limits mixing and favors metabolite retention
Outflow	Venular and meningeal lymphatic pathways	Passive venous oscillations and intrinsic lymphatic pumping mediate ISF efflux and immune drainage	Venous rigidity or lymphatic obstruction impairs clearance and shifts balance toward neuroinflammation
Network-level integration	LC–vascular–glial feedback loops	Coupling of neuromodulatory, mechanical, and hydrodynamic rhythms across hierarchical frequency bands	Optimized during sleep to support efficient clearance; desynchronization at any node disrupts multiscale coupling and increases vulnerability to neurodegeneration

#### The ALPS index

The DTI-ALPS (Analysis Along the Perivascular Space) index has emerged as a robust, indirect marker of glymphatic function. By quantifying water diffusivity along medullary veins and adjacent PVS, ALPS captures the efficiency of perivenous interstitial fluid transport near the lateral ventricles, a key convergence zone for glymphatic inflow and outflow (36, 37). Reduced ALPS values indicate impaired perivascular transport and are frequently accompanied by PVS enlargement and increased extracellular free water.

ALPS alterations have been documented across a broad spectrum of conditions, including Alzheimer’s disease, Parkinson’s disease, cerebral small vessel disease, traumatic brain injury, and normal aging, where progressive ALPS decline parallels PVS dilation and reduced arterial pulsatility (38). Independent studies have confirmed significantly reduced ALPS values in association with impaired perivascular transport and inefficient interstitial clearance (39, 40). When interpreted alongside FW, NODDI, and DKI metrics, ALPS contributes to a multimodal assessment of perivascular and glymphatic pathway integrity (Table 6).

**Table 6:** Strengths and limitations of the DTI-ALPS method.

Strengths	Limitations
Non-invasive and widely accessible, based on conventional DTI acquisitions without contrast agents.	Theoretically deductive framework; the relationship between ALPS index and true human glymphatic function is not fully validated by direct pathophysiological evidence.
Biologically plausible surrogate marker exploiting the anatomical orientation of medullary veins and white matter tracts.	Manual ROI placement introduces subjectivity and limits reproducibility across operators and centers.
Sensitive to physiological aging and multiple neurodegenerative and neuroinflammatory disorders (AD, PD, MS, NMOSD).	Reliance on single-shell DTI (typically b ≈ 1,000 s/mm^2^) prevents separation of diffusion components with different velocities.
Robust for group-level comparisons and longitudinal analyses when standardized protocols are applied.	Measures directional dependence of diffusion rather than diffusion within the perivascular space itself (indirect assessment).
Easily integrated with complementary MRI markers (free-water imaging, PVS burden, microstructural metrics).	Susceptible to partial-volume effects and anatomical distortion, particularly in conditions with brain deformation.
Provides a practical in vivo estimate of perivascular transport efficiency.	Restricted anatomical sampling limited to periventricular white matter; reflects only a regional component of glymphatic function rather than a global measure.

#### Major confounders of glymphatic and ALPS

Consistent with this framework, the ALPS index is reduced in individuals with poor sleep quality or sleep disruption and shows negative associations with choroid plexus volume and sleep disturbance severity, alongside positive associations with global cognitive performance (41, 42). Higher DTI-ALPS values are associated with better sleep quality (lower PSQI scores; *r* = −0.17, *p* = 0.005) and lower cerebral small vessel disease (CSVD) burden (*r* = −0.12, *p* = 0.049) after adjustment for age, sex, vascular risk factors, APOE-ε4 status, and PSMD, with the sleep association remaining significant after further adjustment for median mean diffusivity. Linear regression analyses demonstrate an interaction between CSVD burden and sleep quality, whereby both independently contribute to DTI-ALPS reduction; DTI-ALPS, in turn, relates to multiple cognitive domains and longitudinal MMSE decline and mediates the effects of CSVD burden and sleep quality on cognitive performance, reflecting a synergistic exacerbation of cognitive impairment (43).

In line with evidence linking circadian disruption to glymphatic dysfunction, a multimodal study in night-shift nurses hypothesized impaired glymphatic function measured by DTI-ALPS, associations with downstream neuronal activity changes, links to cognitive, psychological, and sleep-related measures, and involvement of biological processes including circadian regulation, neuroinflammation, and post-translational protein modifications (44).

Vascular factors further modulate glymphatic function: sustained diastolic blood pressure >90 mmHg over 12 years is associated with lower DTI-ALPS values (*β* = −0.038, 95% CI − 0.068 to −0.008), with persistence in individuals aged 45–60 years (45). Consistently, left and mean ALPS indices are reduced in hypertension, and left, right, and mean ALPS indices show negative associations with blood pressure and pulse pressure, linking glymphatic dysfunction to hypertension (46).

In CSVD-related cognitive impairment, reduced global neurovascular coupling and lower cerebral blood flow–to–vascular water content ratios are observed across multiple cortical regions, accompanied by lower ALPS index, increased perivascular space volume fraction, and increased choroid plexus volume. Neurovascular coupling and glymphatic metrics discriminate CSVD-CI from cognitively normal CSVD, and mediation analyses show that ALPS mediates the relationships between neurovascular coupling, cognitive performance, and sleep parameters (47).

Mechanistically, experimental studies demonstrate a causal role of noradrenergic-driven vasomotion in glymphatic transport: α1-adrenergic blockade abolishes vasomotion and CSF influx, whereas locus coeruleus stimulation or α1-agonists restore both. Conversely, zolpidem and propofol suppress locus coeruleus activity and infralow-frequency vasomotion despite preserved slow-wave activity, resulting in ~60% reduction in tracer influx and loss of interstitial metabolite clearance, indicating adrenergic rhythmicity as the primary driver of glymphatic clearance (35).

Additional variability arises from individual physiological factors: linear mixed-effects modeling reveals significant inter-individual variability and interhemispheric asymmetry in enlarged perivascular space load, with increases associated with age, higher total intracranial volume, and lower plasma osmolality (48). Pharmacological modulation of CSF production by acetazolamide, topiramate, GLP-1 receptor agonists, and 11β-HSD1 inhibitors further highlights choroid plexus regulation as a confounder (49). Importantly, most ALPS studies do not control for medications affecting CSF production or vascular tone, sleep quality, circadian phase, blood pressure, hydration status, or cerebrovascular disease burden, complicating interpretation of ALPS-derived metrics.

Most existing ALPS studies do not systematically standardize or control for these factors, which may contribute to variability in the reported findings.

#### Glymphatic assessment using low *b*-value DWI and dual-compartment DTI

Glymphatic function can also be investigated using tailored diffusion protocols. Low *b*-value DWI (5–150 s/mm^2^) enhances sensitivity to slow bulk fluid motion and directional CSF flow within glymphatic pathways. Dual-compartment DTI combines low *b*-value acquisitions sensitive to free-water signal with higher *b*-value acquisitions emphasizing restricted diffusion, enabling separation of extracellular and tissue compartments.

Using this approach (50), demonstrated a diffusivity gradient from the perimesencephalic region toward aqueductal and mesencephalic areas, strongly correlated with free-water content and indicative of altered extracellular fluid distribution. Together, these techniques highlight how diffusion MRI, even at low b-values, can provide physiologically meaningful markers of glymphatic activity and CSF dynamics *in vivo*.

## Multimodal assessment of myelin, microstructure, and connectivity in thalamic and hippocampal regions

### Myelin imaging and microstructural assessment

The evaluation of myelin integrity is a central focus of diffusion MRI research in aging and neurodegenerative diseases. Quantitative myelin imaging increasingly integrates diffusion metrics with complementary approaches, including relaxometry and PET, to improve biological specificity (51). While conventional DTI metrics—such as reduced fractional anisotropy and increased radial diffusivity—are sensitive to demyelination, advanced diffusion models provide greater specificity for axonal packing and myelin–axon interactions. Multidimensional diffusion–relaxation encoding further enhances sensitivity to microstructural complexity in both white matter and deep gray matter, enabling detection of subtle demyelinating changes beyond the limits of classic DTI (1, 11).

### Thalamic microstructure and connectivity

The thalamus is a key hub for sensory integration, cognition, and large-scale brain communication and is highly vulnerable in aging and neurodegeneration. Diffusion MRI studies consistently show progressive thalamic microstructural degeneration—reflected by increased free water, reduced neurite density, and fixel-based abnormalities—closely associated with cognitive impairment. In Alzheimer’s disease, thalamic connectivity loss correlates with amyloid and tau deposition, glymphatic dysfunction, and executive and memory decline (7, 9, 10). Fixel-based analyses further highlight early fiber-specific degeneration in thalamocortical pathways, distinguishing phenotypes and predicting disease progression (8, 52). In Parkinson’s disease, thalamic diffusion abnormalities reflect both neurodegenerative and glymphatic–vascular mechanisms: free-water increases track disease severity and appear linked to impaired glymphatic function (10), while diffusion kurtosis and neurite density imaging reveal localized microstructural breakdown associated with motor and gait impairment (11). Diffusion along perivascular spaces further suggests that impaired CSF clearance contributes to thalamic vulnerability (53).

In demyelinating disorders, the thalamus represents a sensitive biomarker of disease progression. Quantitative MRI studies show early thalamic microstructural degeneration—characterized by reduced neurite density and altered kurtosis metrics—that progresses independently of relapse activity and correlates with cognitive dysfunction, processing-speed impairment, and long-term disability (12, 13, 16, 17, 54).

### Hippocampal microstructure

The hippocampus is predominantly composed of gray matter with a relevant white-matter component. Diffusion tensor imaging has limited ability to resolve hippocampal circuits and microlaminar organization, as FA and mean diffusivity are sensitive but not specific due to laminar averaging effects. Microscopic anisotropy exceeds FA across the hippocampus, reflecting high orientation dispersion, while apparent fiber density provides superior laminar resolution. Biophysical diffusion models, including NODDI-derived intracellular volume fraction, show high sensitivity for hippocampal lamination, myelin content, and longitudinal axis differentiation, and targeted gray-matter models enable assessment of astrocytic and microglial contributions. Ultrafast diffusion times and high angular resolution diffusion MRI further enhance *in vivo* identification of hippocampal circuits (55).

## Artificial intelligence in diffusion MRI for neurodegeneration

Advances in computational power and biophysical modeling have enabled the development of multiple diffusion MRI microstructure models, including the standard model, spherical mean techniques, AxCaliber, diffusion basis spectrum imaging, neurite orientation dispersion and density imaging, white matter tract integrity, SHORE, and soma and neurite density imaging. Despite these methodological advances, the reliable application of artificial intelligence to diffusion MRI microstructure estimation remains challenging, owing to issues related to model complexity, data quality, and clinical generalizability (56).

Within the cognitive impairment spectrum, machine learning approaches have shown potential for classification and risk stratification. Li et al. (57) identified alterations involving the precuneus and cingulate gyrus as relevant features for cognitive classification, reporting high performance when functional and structural connectivity measures were combined within a machine learning framework, with an accuracy of approximately 94% and an area under the receiver operating characteristic curve close to 1.0. Although these results suggest an added value of multimodal connectivity integration, their interpretation remains cautious pending validation in larger and independent cohorts. Similarly, Jiaxuan et al. (58) explored a machine learning approach based on white matter–derived structural and network features to identify individuals at risk of progression from mild cognitive impairment to Alzheimer’s disease. A support vector machine–based white matter signature showed favorable performance, and a joint model integrating imaging features with cognitive scale scores demonstrated improved robustness under cross-validation. Nevertheless, confirmation in well-characterized external cohorts is required before clinical translation.

In the differential diagnosis of neurodegenerative disorders, machine learning and deep learning techniques have demonstrated encouraging results. Mirabian et al. (59) reported that support vector machines and convolutional neural networks, particularly ResNet architectures, yielded promising performance in differentiating frontotemporal dementia from Alzheimer’s disease. However, the authors emphasized that imaging-based models alone cannot fully capture disease complexity, underscoring the necessity of integrating neuroimaging findings with clinical examinations and detailed symptom assessments.

Methodological innovation has also been applied to image enhancement. Yoon et al. (60) proposed a diffusion model–based MRI super-resolution framework that improved diagnostic and prognostic performance in Alzheimer’s disease and mild cognitive impairment. Super-resolved images closely matched higher-field MRI in both image quality and volumetric accuracy and were associated with improved prediction of conversion from mild cognitive impairment to Alzheimer’s disease. Importantly, this study relied on a well-characterized patient cohort with clearly defined inclusion criteria.

Beyond classification tasks, machine learning–based computational approaches have been increasingly applied to investigate cerebrovascular and glymphatic alterations in Alzheimer’s disease. Fatima et al. (61) integrated imaging and behavioral data to explore vascular structure, fluid transport dynamics, and potential early markers of glymphatic dysfunction. While these approaches offer novel insights into vascular–glymphatic interactions, additional validation is required to clarify causality and clinical relevance.

In movement disorders, artificial intelligence has shown particular promise in differential diagnosis. Vaillancourt et al. (62) developed and validated the multicenter Automated Imaging Differentiation of Parkinsonism framework in rigorously selected cohorts of patients with Parkinson’s disease, multiple system atrophy, and progressive supranuclear palsy. The model demonstrated excellent discriminative performance, with areas under the receiver operating characteristic curve (AUCs) of 0.96 for PD versus atypical parkinsonism, 0.98 for MSA versus PSP, 0.98 for PD versus MSA, and 0.98for PD versus PSP. These consistently high AUC values indicate robust classification accuracy across all pairwise diagnostic comparison. The model demonstrated excellent discriminative performance, with areas under the receiver operating characteristic curve (AUCs) of 0.96 for PD versus atypical parkinsonism, 0.98 for MSA versus PSP, 0.98 for PD versus MSA, and 0.98for PD versus PSP. These consistently high AUC values indicate robust classification accuracy across all pairwise diagnostic comparison.

More broadly, artificial intelligence and machine learning offer unprecedented capacity to integrate large, multidimensional datasets, including longitudinal digital biomarkers derived from wearable sensors, sleep physiology, neuroimaging, and electronic health records. These approaches have demonstrated the ability to detect subtle preclinical motor and non-motor changes, particularly in Parkinson’s disease. However, clinical deployment remains limited by regulatory, methodological, and validation challenges. Without rigorous validation, transparent model development, and representative datasets, AI-based tools risk amplifying bias rather than reducing diagnostic uncertainty.

At a mechanistic level, artificial intelligence has also been proposed as a means to uncover convergent biological pathways linking oxidative stress, neurodegeneration, and metabolic disease. While such integrative approaches may reveal interactions beyond the reach of traditional statistical methods, they remain at an early developmental stage and require substantial validation before clinical application (63).

In multiple sclerosis, deep learning approaches have been explored for both structural and functional biomarkers. Skattebøl et al. (64) demonstrated that deep learning–based brain age estimation is feasible and significantly associated with clinical disability in a well-characterized community-based cohort. Retinal imaging studies further highlight the potential of artificial intelligence: Farabi Maleki et al. (65) reviewed evidence showing that machine learning and deep learning enhance optical coherence tomography–based detection, segmentation, and classification of retinal abnormalities, supporting disease monitoring and prognostication. Nevertheless, ethical, methodological, and translational challenges remain critical barriers to widespread implementation.

At the same time, caution is warranted when interpreting exceptionally high classification performance. Ekmekyapar et al. (66) reported near-perfect accuracy using a hybrid deep learning and feature-based framework for multiple sclerosis classification; however, limited reporting on cohort composition, data provenance.

Complementary methodological perspectives are provided by radiomics and connectomics, which together enable the integration of local microstructural alterations and large-scale network organization. When combined, these approaches offer a powerful framework for linking tissue-level changes to connectivity and functional disruption (67).

Finally, emerging strategies seek to address fundamental limitations in training data and ground-truth availability. Fang (68) proposed the use of physics-informed synthetic data to train supervised models for diffusion MRI microstructure estimation, while Liao et al. (69) demonstrated that machine learning–based multicompartment diffusion models can capture axonal density, orientation, and integrity across development, acute ischemia, and multiple sclerosis. Although these approaches show broad applicability, their reliance on machine learning underscores the importance of training dataset selection, uncertainty quantification, and external validation.

NODDI capture subtle microstructural alterations in brain GM and WM, demonstrating advantages over standard DT imaging in capturing disease-relevant alterations. By integrating NODDI with cognitive data, machine-learning models can learn complex patterns and relationships facilitating the differentiation of FTD subtypes (70).

External validation is essential to assess the robustness of AI-based diffusion MRI models, as domain shift related to scanner differences, acquisition protocols, and population characteristics may substantially affect performance. In addition, fairness considerations are critical, since models trained on non-representative datasets may exhibit biased performance across demographic or clinical subgroups, underscoring the need for multi-center and diverse validation cohorts.

AI models trained on data from specific scanners, acquisition protocols, or patient populations may not generalize reliably to other hospitals or clinical settings. Therefore, careful multi-center validation, ongoing performance monitoring, and strategies to mitigate overfitting and overconfident predictions are necessary before clinical deployment.

## Clinical application

Table 7 provides a comparative and clinically oriented overview of diffusion MRI–based biomarkers, highlighting their potential roles, practical feasibility, and main limitations rather than implying a hierarchical ranking or definitive diagnostic utility.

**Table 7:** Comparative overview of diffusion MRI–based biomarkers in neurodegenerative diseases.

Technique/ biomarker	Main clinical contexts	Diagnostic/prognostic role*	Invasiveness	Cost and availability	Strengths	Key limitations	Level of evidence^†^
Mac word desktop	AD, PD, aging, prodromal stages	Early stratification; longitudinal progression	Non-invasive	Low–moderate; widely available	Robust across scanners; sensitive to neurodegeneration and aging	Indirect microstructural proxy; influenced by vascular and inflammatory factors	Moderate–High
Fixel-Based Analysis (FBA)	AD (early vs. late-onset), aging	Detection of tract-specific WM degeneration	Non-invasive	Moderate; requires advanced processing	Resolves crossing fibers; higher specificity than DTI	Higher technical complexity; limited standardization	Moderate
NODDI/multi-compartment models	AD, aging, PD, MS	Early microstructural alterations; differential sensitivity	Non-invasive	Moderate; increasing availability	Greater biological interpretability than DTI	Model assumptions; acquisition demands	Moderate
ALPS index	AD, PD, MS, iRBD	Glymphatic dysfunction; progression risk	Non-invasive	Low; standard diffusion MRI	Simple implementation; pathophysiological insight	Strongly confounded (sleep, BP, CSVD, hydration); indirect surrogate	Low–Moderate
Perivascular space (PVS)–related metrics	AD, AD+CSVD	Vascular–glymphatic interaction	Non-invasive	Low; conventional MRI	Multimodal consistency (PET, CSF, plasma)	Limited specificity; segmentation variability	Moderate
Network/connectomic metrics	AD, PD, MS	Systems-level degeneration patterns	Non-invasive	Moderate–high; specialized pipelines	Captures distributed pathology	False positives; multiple comparisons; tractography bias	Low–Moderate
Multimodal diffusion + PET/CSF/plasma	AD, PD	Risk stratification; disease staging	Minimally invasive (biofluids)	High; limited availability	Biological validation; improved specificity	Cost; limited scalability	High
AI-based diffusion models	AD, PD, MS	Classification; prognostic enrichment	Non-invasive	Variable; limited clinical deployment	High-dimensional integration	Overfitting; domain shift; limited external validation	Low–Moderate

*Clinical roles are indicative and context-dependent; diffusion MRI biomarkers are currently complementary rather than stand-alone tools. ^†^Level of evidence reflects consistency across cohorts, use of reference standards, and external validation.

### Aging

Healthy aging is associated with progressive microstructural, fluid-dynamic, and network-level brain changes detectable with diffusion MRI. These include declining white-matter integrity, characterized by reduced anisotropy and increased diffusivity, fiber-specific axonal loss and tract shrinkage, and gradual simplification of neuronal and neuritic architecture. In parallel, gray-matter microstructural complexity decreases, while extracellular free water and perivascular space burden increase, reflecting impaired interstitial fluid homeostasis and glymphatic transport. Age-related reductions in the ALPS index further indicate declining perivascular clearance efficiency. At the network level, diffusion-based connectomics reveals reduced global efficiency and preferential vulnerability of limbic and default-mode network hubs. Together, these diffusion-derived markers define a physiological aging signature that provides a critical reference framework for distinguishing normal aging from early neurodegenerative trajectories. In the interpretation of the results, vascular risk factors, metabolic conditions, and frailty—as detailed in the *Confounders of Brain Aging* section—were considered as key potential confounders, given their known influence on diffusion MRI metrics, brain structure, and network integrity (Table 8).

**Table 8:** Diffusion MRI correlates of physiological brain aging.

Domain	Diffusion MRI findings	Biological interpretation	Key evidence
White matter integrity (DTI)	Decreased fractional anisotropy with increased mean, axial, and radial diffusivity in associative and commissural tracts	Myelin rarefaction (↑ radial diffusivity) and early axonal cytoskeletal instability (↑ axial diffusivity)	(90)
Fiber-specific degeneration (FBA)	Reduced fiber density and fiber cross-section, particularly in the fornix and forceps minor	Combined axonal loss and tract shrinkage, associated with cognitive decline	(91)
Neuronal soma microstructure (multicompartment models)	Reduced soma signal fraction and soma radius with increased extracellular diffusivity	Neuronal shrinkage and simplification of cortical cytoarchitecture	(92, 93)
Neurite architecture (NODDI / neurite models)	Reduced neurite density and increased orientation dispersion in white and gray matter	Dendritic and axonal disorganization with extracellular space expansion	(93)
Gray matter microstructure (DKI)	Decreased mean and radial kurtosis in temporoparietal and cingulate cortices	Loss of microstructural complexity preceding overt cortical atrophy	(8)
Extracellular fluid compartment (free-water imaging)	Increased free-water fraction, especially in periventricular and frontal white matter	Extracellular expansion and impaired interstitial fluid clearance	(15, 38)
Perivascular spaces (PVS)	Age-related enlargement of PVS in the centrum semiovale and basal ganglia	Structural remodeling of perivascular pathways and reduced clearance efficiency	(32, 94)
Longitudinal aging effects	Higher baseline PVS burden associated with accelerated white matter degeneration over time	PVS-mediated contribution to longitudinal white matter damage, potentially modulated by sleep	(95)
Glymphatic function (DTI-ALPS)	Progressive age-related reduction in ALPS index	Decline in perivascular and glymphatic transport related to vascular stiffening and aquaporin-4 alterations	(38)
Early aging glymphatic alterations	Detectable age-related changes in ALPS index and free-water fraction already in early older adulthood; interrelated changes with choroid plexus volume	Early glymphatic inefficiency contributing to normal aging pathophysiology	(96)
Structural connectivity (connectomics)	Reduced global efficiency, posterior-to-anterior disconnection, and decreased interhemispheric connectivity	Vulnerability of default mode and limbic hubs supporting cognition	(8, 91)

### Mild cognitive impairment (MCI)

Mild Cognitive Impairment represents an intermediate stage between healthy aging and Alzheimer’s disease, characterized by early microstructural, fluid-dynamic, and network-level alterations detectable with diffusion MRI. White-matter degeneration affects limbic, hippocampal–thalamic, and associative pathways and is accompanied by early gray-matter neuritic loss in hippocampal and posterior cortical regions. In parallel, extracellular free-water expansion and early glymphatic dysfunction indicate impaired interstitial fluid regulation before overt dementia. Disruption of structure–function coupling and diffuse white-matter hyperintensity–related injury further contribute to network inefficiency, highlighting MCI as a phase in which microstructural damage, clearance failure, and partial compensatory remodeling coexist and influence the risk of progression to Alzheimer’s disease. In the interpretation of the results, vascular risk factors, metabolic conditions, and frailty—as detailed in the Confounders of Brain Aging section—were considered as key potential confounders, given their known influence on diffusion MRI metrics, brain structure, and network integrity (Table 9).

**Table 9:** Diffusion MRI–derived microstructural and fluid-dynamic features of mild cognitive impairment (MCI).

Domain	Diffusion MRI findings	Pathophysiological interpretation	Key references
Disease trajectory	FA and diffusivity metrics predict conversion to AD (≈10–12%/year)	Early WM microstructural damage as marker of progression risk	(58)
White-matter tracts (DTI)	↓ FA; ↑ MD, AD, RD in cingulum, fornix, uncinate fasciculus, splenial fibers	Early degeneration of limbic, hippocampal–thalamic, and associative pathways	(97, 98)
Fiber-specific degeneration (FBA)	↓ Fiber density and fiber cross-section in limbic/DMN tracts	Axonal degeneration affecting the structural backbone of the DMN	(29, 99)
Gray-matter microstructure (NODDI)	↓ Neurite density; altered orientation dispersion in hippocampus, PCC, precuneus	Dendritic regression and synaptic simplification	(100, 101)
Cortical and superficial WM integrity	Diffusion abnormalities extending into cortex and SWM	Microstructural injury preceding volumetric atrophy	(87)
Extracellular fluid (FW imaging)	↑ Free-water fraction, especially posterior WM	Early interstitial fluid dysregulation linked to amyloid pathology	(29)
Glymphatic function (DTI-ALPS)	Left-hemispheric ↓ ALPS index	Early impairment of perivascular/glymphatic transport	(23)
Structure–function coupling	Reduced coupling between structural and functional networks	Network inefficiency linked to microstructural and clearance failure	(23)
White-matter hyperintensities (WMH)	WMH-associated microstructural damage extending beyond visible lesions	Diffuse extralesional injury with partial compensatory remodeling	(102, 103)
Network-level effects	↓ Global connectivity with focal frontal/limbic increases	Early network disconnection with compensatory reorganization	(103)

### Alzheimer’s disease (AD)

Alzheimer’s disease is characterized by a highly reproducible constellation of microstructural, fluid-dynamic, and network-level abnormalities detectable with diffusion MRI. Early gray-matter microstructural degeneration affects the medial temporal lobe, with diffusion kurtosis and neurite-based models revealing loss of microstructural complexity, neurite density reduction, and extracellular expansion that often precede overt atrophy. White-matter degeneration involves limbic and associative tracts critical for memory and executive function, with fiber-specific analyses demonstrating axonal loss and tract shrinkage. At the network level, diffusion connectomics highlights preferential vulnerability of default-mode network hubs, consistent with connectivity-constrained propagation of tau pathology. In parallel, profound alterations in brain fluid homeostasis emerge across the disease continuum, including reduced glymphatic transport, perivascular space enlargement, and marked free-water expansion. These changes correlate with amyloid and tau burden, astroglial dysfunction, and cognitive decline, supporting a model in which impaired clearance mechanisms interact with microstructural degeneration to drive disease progression (Table 10).

**Table 10:** Integrated diffusion MRI markers of glymphatic dysfunction and neurodegeneration across the Alzheimer’s disease continuum.

Domain	Key diffusion MRI findings	Pathophysiological interpretation	Key references
Gray-matter microstructure (DKI, NODDI)	↓ Mean and radial kurtosis; ↓ neurite density; ↑ isotropic volume fraction in hippocampus, entorhinal and parahippocampal cortex	Synaptic loss, dendritic regression, tau-mediated cytoskeletal destabilization preceding atrophy	(8, 100, 101, 104)
White-matter microstructure (DTI, FBA)	Degeneration of fornix, cingulum, uncinate fasciculus, SLF; ↓ FA; ↑ MD/RD; ↓ fiber density and fiber cross-section	Axonal degeneration and tract atrophy disrupting limbic and default-mode networks	(29, 31, 99)
Fiber architecture (FBA)	Detection of tract-specific degeneration not captured by tensor metrics	Improved biological specificity for axonal loss and tract shrinkage	(29)
Network organization (connectomics)	↓ Nodal efficiency and clustering; preferential DMN hub involvement	Network-level disconnection consistent with trans-synaptic tau propagation (Braak-like pattern)	(31, 103)
Glymphatic function (DTI-ALPS)	Progressive reduction of ALPS index from MCI to AD; often left-hemispheric predominance	Early and progressive impairment of perivascular–glymphatic transport predicting amyloid burden, atrophy, and cognitive decline	(32, 36–38)
Perivascular spaces (PVS)	Increased PVS volume in white matter and basal ganglia	Structural remodeling of perivascular pathways reflecting impaired waste clearance	(32, 33, 94)
Free-water dynamics (FWI)	↑ Extracellular free water in GM and WM, including choroid plexus; larger effect sizes than MD/RD	Extracellular expansion, BBB-related neuroinflammation, impaired CSF–ISF turnover	(23, 27, 37)
Choroid plexus FW	↑ FW in choroid plexus	Impaired CSF production and turnover; predictor of faster cognitive decline	(105)
Astroglial mechanisms	ALPS reduction associated with FW increase, PVS enlargement, WMH burden, ↑ GFAP, AQP4 depolarization	Failure of the neurovascular unit with astrocytic stress and impaired clearance	(105, 106)
White-matter hyperintensities (WMH)	PVS and FW abnormalities associated with WMH burden	Perivascular edema and diffuse white-matter injury	(33)
Early PVS biomarker	Centrum semiovale PVS enlargement without CSF or lifestyle associations	PVS dysfunction as early imaging marker of AD	(107)
Clinical correlates	Diffusion abnormalities correlate with memory, executive, and attentional deficits	Integrated microstructural degeneration and clearance failure drive cognitive decline	(32, 106)
ALPS/Aβ42/40	ALPS–cognition associations remain significant after adjustment for age, sex, plasma A*β*42/40	Robust methodological support for glymphatic markers	(108)

### Parkinson’s disease (PD)

Parkinson’s disease is a multisystem neurodegenerative disorder whose clinical expression extends beyond its classical motor phenotype. Increasing evidence shows that non-motor symptoms often precede motor onset and substantially contribute to disease burden, disability, and reduced quality of life, reflecting early and sustained dysfunction across autonomic, cognitive, affective, sensory, and brainstem networks.

In this context, advanced diffusion MRI reveals widespread microstructural, fluid-dynamic, and network-level alterations involving limbic, thalamic, associative, brainstem, and autonomic pathways, frequently detectable before overt motor symptoms. Neurite-based and kurtosis diffusion models identify gray- and white-matter degeneration associated with both motor and non-motor manifestations and support phenotypic distinctions such as brain-first and body-first Parkinson’s disease. Concurrently, increased free water, perivascular space enlargement, and progressive reduction of the DTI-ALPS index indicate impaired glymphatic and perivascular clearance linked to *α*-synuclein pathology, sleep disturbances, cognitive decline, and dementia conversion.

Collectively, these findings support a shift from dopamine-centric models toward a systems-level, network-based view of Parkinson’s disease, positioning diffusion MRI as an integrated framework to capture microstructural degeneration, network vulnerability, and clearance failure across the disease spectrum—processes that drive clinical heterogeneity yet remain underrepresented by motor-focused assessments (Tables 11, 12) (71).

**Table 11:** Integrated diffusion MRI markers of microstructural and glymphatic dysfunction in Parkinson’s disease.

**Domain**	**Key diffusion MRI findings**	**Clinical/pathophysiological implications**	Key references
Glymphatic function (DTI-ALPS)	Global reduction of ALPS index; left-hemispheric predominance; progressive decline with disease severity	Impaired perivascular–glymphatic clearance contributing to α-synuclein accumulation, cognitive vulnerability, and risk of Parkinson’s disease dementia (PDD)	(109–111)
Perivascular spaces (PVS/EPVS)	Increased EPVS, particularly in basal ganglia; inverse correlation with ALPS values	Structural remodeling of perivascular pathways, fluid stagnation, impaired clearance, and possible tau co-pathology	(109, 110, 112)
Free-water dynamics (FWI)	Increased free water in substantia nigra detectable in prodromal and early PD; extension to limbic and thalamic pathways	Marker of nigrostriatal degeneration, neuroinflammation, and glymphatic dysfunction; predictor of disease progression and cognitive decline	(113–117)
Nigrostriatal microstructure (NODDI, DKI)	Reduced intracellular volume fraction and altered orientation dispersion in SNpc, typically contralateral to symptoms; increased basal-ganglia kurtosis	Axonal and dendritic degeneration underlying motor impairment and early basal-ganglia involvement	(118–120)
Limbic and paralimbic circuits	Early diffusion abnormalities in amygdala and medial temporal lobe	Explains non-motor symptoms (cognitive impairment, anxiety, depression); differentiates brain-first vs. body-first PD phenotypes	(118)
Thalamic and thalamocortical pathways	Decreased neurite density; increased free water; altered orientation dispersion in thalamic relay nuclei and projections	Executive, attentional, and visuospatial deficits; thalamus as a central glymphatic and network hub	(116, 119, 120)
White-matter networks (DTI, FBA)	Degeneration of frontostriatal and associative tracts (↓ FA, ↑ RD; ↓ fiber density and fiber cross-section)	Network disconnection facilitating trans-synaptic *α*-synuclein propagation and cognitive dysfunction	(117, 121)
Autonomic and brainstem pathways	Diffusion abnormalities in dorsal motor nucleus of the vagus, nucleus ambiguus, and sympathetic tracts	Supports peripheral/autonomic origin of PD in a subset of patients; explains prodromal autonomic symptoms	(118)
Sleep–glymphatic axis	Accelerated ALPS decline and free-water expansion in patients with sleep disorders (e.g., REM sleep behavior disorder)	Sleep-related glymphatic impairment amplifies extracellular fluid accumulation and accelerates disease progression	(109, 122, 123)
Differential diagnosis	Distinct free-water and neurite-based diffusion patterns compared with AD and atypical parkinsonian syndromes	Disease-specific proteinopathies and clearance mechanisms; improved diagnostic stratification	(113, 114)

**Table 12:** DTI-ALPS as an integrative biomarker of glymphatic dysfunction in Parkinson’s disease.

Category	Key findings	Clinical/pathophysiological implications	Authors
Global ALPS reduction	ALPS significantly reduced in PD vs. controls	Global glymphatic dysfunction	(112, 116, 117, 122)
α-synuclein pathology	ALPS decline parallels α-syn accumulation	Links clearance failure to core PD proteinopathy	(112, 116, 117)
Motor severity	Lower ALPS correlates with motor impairment	Glymphatic dysfunction contributes to motor decline	(112, 116)
Cognitive decline	Lower ALPS associated with executive/cognitive deficits	Marker of cognitive vulnerability	(109, 110)
Sleep disturbances	Sleep disorders → faster ALPS decline	Sleep–glymphatic axis in PD progression	(116, 123)
Age effects	Stronger ALPS reduction in patients <65 years	Age-dependent pathogenic mechanisms	(116)
Dementia conversion (PDD)	Low ALPS + ↑ PVS predict PDD	Biomarker of dementia risk	(110)
PVS–ALPS relationship	EPVS inversely correlated with ALPS	Impaired clearance; tau co-pathology	(109)
CSF / lymphatic dynamics	↓ CSF mobility, ↓ meningeal drainage, ↓ AQP4	Global glymphatic–perivascular failure	(111)
Phenotypic variants	ALPS differs in brain-first vs. body-first PD	Distinct α-syn propagation routes	(117)

### Multiple sclerosis (MS)

Multiple sclerosis is now recognized as a diffuse neurodegenerative disorder rather than a purely focal inflammatory demyelinating disease. Advanced diffusion MRI has demonstrated widespread microstructural damage involving normal-appearing white matter, cortical and deep gray matter, and large-scale white-matter networks. These alterations reflect combined demyelination, axonal degeneration, glial dysfunction, and neuronal injury, and correlate closely with disability progression, cognitive impairment, and network disruption. In addition, growing evidence indicates a role for perivascular, blood–brain barrier, and glymphatic dysfunction, with reduced DTI-ALPS index, free-water expansion, and perivascular space enlargement emerging as markers of disease severity and progression, particularly in progressive MS phenotypes. Multiple sclerosis is associated with vascular risk factors and widespread neurovascular pathology. Several cellular components of the neurovascular unit become dysfunctional in multiple sclerosis; however, it remains unclear whether dysfunction of key cell types—including endothelial cells, pericytes, and astrocytes—represents a primary driver or a secondary consequence of disease pathophysiology (Table 13) (72).

**Table 13:** Diffusion MRI in Multiple Sclerosis.

Domain	Key diffusion MRI findings	Pathophysiological interpretation	Key references
Normal-appearing white matter (DTI)	↓ FA; ↑ MD and RD in NAWM beyond visible lesions	Early demyelination and axonal disorganization preceding macroscopic atrophy	(28, 124)
Diffuse extra-lesional degeneration	Widespread diffusion abnormalities outside focal plaques	MS as a diffuse neurodegenerative disorder rather than a purely focal inflammatory disease	(28)
Gray-matter microstructure (DKI)	↓ Mean and radial kurtosis in cortex	Dendritic pruning, synaptic loss, microstructural disorganization preceding cortical atrophy	(30)
Gray-matter microstructure (NODDI)	↓ Neurite density; altered orientation dispersion in cortex, thalamus, hippocampus, deep GM nuclei	Neuronal and dendritic loss contributing to cognitive impairment	(125, 126)
Lesion microstructure – acute lesions	↓ FA; ↑ diffusivities; ↓ intracellular volume fraction; ↑ extracellular volume and free water; periplaque extension	Acute inflammation, BBB disruption, cellular infiltration with tissue injury extending beyond lesion core	(127, 128)
Lesion microstructure – chronic inactive lesions	Persistently ↓ neurite density; ↑ RD	Permanent axonal and myelin loss, particularly in progressive MS	(127)
Chronic active/paramagnetic rim lesions	Severe neurite loss; marked ↑ free water; microstructural rim abnormalities	Smoldering inflammation with iron-laden microglia and ongoing axonal destruction	(127)
White-matter tract degeneration (NODDI, FBA)	↓ Neurite density; ↓ fiber density and fiber cross-section in cingulum, corpus callosum, SLF, thalamic radiations	Axonal degeneration and tract atrophy disrupting cognitive networks	(77, 129)
Network organization (structural connectomics)	Reduced global efficiency and tract-specific network disruption	Large-scale disconnection underlying cognitive slowing and impairment	(77)
Free-water dynamics (FWI)	Global ↑ extracellular free water across WM, GM, and NAWM	Chronic inflammation, BBB dysfunction, extracellular fluid expansion	(29, 130)
Thalamic microstructure (multimodal diffusion)	↓ Neurite density; abnormal kurtosis metrics; ↑ free water; early disruption of thalamic radiations	Vulnerability of thalamus as central relay hub driving cognitive and disability progression	(30, 77)
Perivascular/glymphatic function (DTI-ALPS)	↓ ALPS index across MS stages; greater reduction in progressive forms	Impaired perivascular–glymphatic clearance associated with inflammation and neurodegeneration	(39, 131)
Disease progression (RRMS → SPMS)	Further ALPS reduction during transition to SPMS	Early biomarker of progression linked to clearance failure	(39)
BBB and perivascular pathology	↑ Free water; ↑ ADC in acute plaques; PVS-related extracellular expansion	Vasogenic edema, BBB breakdown, chronic perivascular inflammation	(30, 127, 128)
Clinical correlates	Diffusion abnormalities correlate with physical disability, cognitive decline, and processing speed	Integrated axonal loss, GM degeneration, and clearance dysfunction drive clinical worsening	(28, 77)

### Neuromyelitis optica spectrum disorder (NMOSD): diffusion MRI evidence of astrocytopathy

#### Neuromyelitis optica spectrum disorder

Overall, diffusion MRI evidence indicates that NMOSD is characterized by widespread microstructural and fluid-dynamic alterations extending beyond focal lesions, in line with a primary astrocytopathy. Abnormalities across white and gray matter, deep gray nuclei, and perivascular compartments support a diffuse pattern of tissue disorganization rather than isolated tract damage. The consistent involvement of extracellular and free-water compartments, together with alterations in neurite-related metrics, is compatible with impaired astrocytic water regulation and disrupted glio-vascular coupling. Diffusion-based markers of perivascular and glymphatic function further suggest a marked clearance dysfunction, often reported as more pronounced than in multiple sclerosis. Collectively, these findings support diffusion MRI as a sensitive *in vivo* approach to capture the systemic impact of astrocytic injury in NMOSD, while underscoring the need for longitudinal and multimodal validation (Table 14).

**Table 14:** Integrated diffusion MRI markers of microstructural, perivascular, and glymphatic dysfunction in neuromyelitis optica spectrum disorder (NMOSD).

Domain	Key diffusion MRI findings	Pathophysiological interpretation	Key references
Primary astrocytic pathology	Diffuse reductions in neurite density (NDI) and increased isotropic diffusion (ISOVF) in lesions, NAWM, cortex, and deep gray matter	Autoimmune AQP4-IgG–mediated astrocytic destruction with loss of water-regulation capacity and glio-vascular coupling	(132, 133)
Extracellular and free-water dynamics	Marked free-water expansion across WM, GM, optic radiations, and thalamus	Extracellular space enlargement driven by astrocytic failure rather than inflammatory edema	(128, 133)
Gray-matter microstructure	Reduced mean and radial kurtosis; decreased NDI in cortex and deep gray nuclei	Dendritic simplification, synaptic loss, and microstructural complexity reduction preceding macroscopic atrophy	(134, 135)
Deep gray matter vulnerability	↑ MD, ↓ FA, ↑ FW, ↓ NDI in thalamus, basal ganglia, hypothalamus	Loss of astrocytic metabolic support in highly connected hubs leading to network-level dysfunction	(132, 133)
White-matter tracts	Reduced fiber density and fiber cross-section in optic radiations, corticospinal tracts, and thalamocortical pathways	Secondary axonal degeneration following primary astrocytic injury, paralleling visual and motor disability	(132, 133)
Glymphatic function (DTI-ALPS)	Significantly reduced ALPS index, often more severe than in MS	Profound impairment of CSF–interstitial fluid exchange due to AQP4 loss at astrocytic endfeet	(39, 136)
Perivascular spaces (PVS)	PVS enlargement with abnormal diffusion, especially in centrum semiovale	Perivascular edema, BBB disruption, and stagnation of interstitial solute clearance	(135, 136)
Blood–brain barrier and inflammation	FW increase and diffusion abnormalities linked to BBB leakage and complement-mediated injury	Amplification of neuroinflammation and secondary neurodegeneration	(128, 135)
Clinical correlations	Diffusion abnormalities correlate with visual impairment, motor disability, cognitive decline	Diffusion MRI metrics as sensitive biomarkers of severity and spatial distribution of NMOSD damage	(132, 133)

## Diffusion MRI biomarkers

### Voxelwise and Network-Level Diffusion MRI Evidence

Voxelwise diffusion MRI approaches, including tract-based spatial statistics (TBSS), voxel-based morphometry, and advanced diffusion modeling, have been widely applied to characterize white and gray matter microstructural alterations across metabolic, neurodegenerative, inflammatory, and movement disorders. These methods enable unbiased whole-brain assessment of disease-related changes and have increasingly been combined with longitudinal designs, machine learning, and network-level analyses to improve sensitivity and interpretability. The following table summarizes key voxelwise and TBSS-based studies, highlighting imaging techniques, main findings, and clinical relevance (Table 15).

**Table 15:** Summary of voxelwise/TBSS studies.

Study	Population	Imaging methods	Main findings	Clinical/pathophysiological implications
(100)	Type 2 diabetes mellitus	TBSS, ALPS index	Reduced ALPS index associated with lower cognitive performance	Suggests glymphatic-related white matter alterations linked to cognition
(137)	RRMS vs. NMOSD	TBSS, ROI, free-water imaging, DTI	Increased WM water content in RRMS; FW changes more limited than DTI alterations	Highlights differential sensitivity of FW vs. conventional DTI
(133)	MRI-positive NMOSD	TBSS, DTI	Diffuse WM abnormalities across all DTI metrics	Supports widespread WM involvement in NMOSD
(138)	Mild cognitive impairment	TBSS meta-analysis	Widespread FA reductions in corpus callosum and left striatum; correlations with age and MMSE	Links callosal microstructural damage to aging and cognitive decline
(139)	MCI → AD (longitudinal)	Longitudinal DTI, TBSS	Progressive increases in AD and RD; AD changes correlated with cognitive decline	Axial diffusivity as a marker of disease progression
(140)	Alzheimer’s disease	TBSS, multi-metric DTI, SVM	CC, CR, SLF most affected; FA most informative metric	TBSS + ML improves classification; validation needed
(141)	AD vs. LBD vs. HC	TBSS, DTI	Reduced FA and AD, increased RD across major WM tracts; early frontal GM involvement	Detectable WM and GM changes in early AD
(142)	Parkinson’s disease	TBSS, network analysis	WM alterations in prefrontal, callosal, thalamic tracts; correlations with motor and non-motor symptoms	Network disorganization linked to clinical severity
(120)	Parkinson’s disease	Voxelwise DKI, ML	Global GM microstructural impairment; CSTC circuit disruption	DKI supports PD–HC differentiation
(143)	MSA-P vs. PD vs. HC	TBSS, FW, MT-sat	Diffuse WM alterations in MSA-P; MCP differentiates MSA-P from PD	Distinct microstructural signatures in atypical parkinsonism
(144)	MCI+	VBM, TBSS	WM connectivity loss in Papez circuit may precede temporal GM atrophy	WM alterations as early markers in prodromal AD
(27)	Alzheimer’s disease	VBM, GM-based spatial statistics, FW, DTI	Multimodal voxelwise metrics detect AD-related GM changes	Supports multimodal voxelwise assessment of AD pathology

### Network construction and statistical analysis

Brain networks were constructed using predefined anatomical or functional parcellation schemes. Nodes represented brain regions, while edges corresponded to pairwise relationships derived from imaging-based connectivity measures. Subject-specific connectivity matrices were generated and preprocessed to reduce noise and spurious connections.

Node-wise analysis focused on topological properties of individual nodes, including degree, strength, betweenness centrality, clustering coefficient, and nodal efficiency. Group-level comparisons of node-wise metrics were performed using appropriate statistical tests, with multiple comparisons controlled at the node level using false discovery rate (FDR) correction.

Edge-wise analysis directly compared individual connections between node pairs across groups or conditions. Given the high dimensionality of connectomic data, mass-univariate edge-wise testing poses a substantial multiple comparison problem. To address this issue, network-based statistics (NBS) were employed to control the family-wise error rate (FWER) at the subnetwork level, retaining only statistically significant connected components rather than isolated edges.

To enhance robustness, connectivity matrices were thresholded using proportional or statistically informed criteria, and denoising and regularization techniques were applied. Sensitivity analyses were conducted across different thresholds and parcellation schemes. Reproducibility was assessed through cross-validation and stability analyses. For improved interpretability, edge-wise findings were summarized at the node and subnetwork levels, combining the sensitivity of edge-wise approaches with the robustness of node-wise metrics.

### Multiple comparisons and exploratory analyses

Both voxelwise and connectomic neuroimaging analyses involve a very large number of simultaneous statistical tests, substantially increasing the risk of false positive findings. In diffusion MRI and structural connectomic studies, this high dimensionality requires careful control of type I error rates. While voxelwise analyses benefit from spatial correlations that can be exploited using permutation-based inference and threshold-free cluster enhancement, connectivity matrices exhibit weaker spatial structure, making conventional correction strategies overly conservative.

Network-based approaches address this limitation by aggregating statistically associated edges into connected subnetworks, improving statistical power while maintaining FWER control. However, these methods often rely on user-defined cluster-forming thresholds, introducing an element of arbitrariness and motivating the development of adaptive and threshold-free network inference frameworks.

Exploratory analyses are frequently adopted in connectome studies due to the limited availability of strong *a priori* hypotheses regarding the size, location, and topology of disease-related subnetworks. While essential for hypothesis generation, exploratory analyses are associated with increased risks of overfitting and inflated effect sizes. Accordingly, exploratory findings were interpreted cautiously, clearly distinguished from confirmatory analyses, and evaluated using cross-validation or independent validation strategies where possible.

Group-level brain connectome analysis plays an increasingly important role in neuropsychiatric research for identifying disease-related subnetworks, but remains challenged by limited prior knowledge of subnetwork size and location and by substantial noise in neuroimaging data. To address these issues, Wu et al. (73) proposed a likelihood-based adaptive dense subgraph discovery (ADSD) model that enables robust identification of disease-related subnetworks while accounting for both false-positive and false-negative errors in edge-wise inference.

Statistical power estimation represents an additional challenge in connectome studies. Bi et al. (74) introduced *BNPower*, a toolkit for power analysis in brain network research, highlighting difficulties related to network complexity, multidimensional effect size definition, and family-wise error rate (FWER) control in high-dimensional graph data. The proposed framework incorporates graph ℓ₀-shrinkage–based methods and permutation testing to improve feasibility and reliability.

Network-based methodologies have also been applied to structural MRI to enhance the specificity of neurodegeneration biomarkers. He et al. (75) proposed a whole-brain network analysis of volumetric MRI changes using a multiple random eigengraph framework and multigraph embedding, enabling identification of spatially distributed neurodegeneration patterns in Alzheimer’s disease while controlling for confounders and FWER.

Functional connectivity alterations have been explored using signal-based approaches, such as the tensor product coupling method applied to whole-brain cross-frequency coupling, which revealed connectivity differences associated with Alzheimer’s disease (76). In structural connectivity studies, Martínez-Heras et al. (77) combined advanced tractography, graph-theoretical measures, and machine learning, showing fewer network alterations in primary progressive multiple sclerosis compared with other disease phenotypes.

Finally, multimodal network-informed approaches have been proposed to characterize neurodegeneration. Mondragon et al. (78) demonstrated convergent assessment of locus coeruleus integrity using PET and MRI, alongside exploratory associations with cognitive and neuropsychiatric measures, supporting the potential of multimodal connectomic biomarkers while underscoring the need for longitudinal validation.

Many published diffusion MRI findings are not consistently corrected for all tested metrics, regions, or network nodes, and as a result, some reported voxelwise or connectomic alterations may represent false-positive findings rather than robust biological effects.

### Confounders of brain aging

Vascular risk factors, metabolic disease, and frailty substantially influence diffusion MRI metrics and may account for a significant proportion of the changes often attributed to “healthy aging” or early neurodegenerative processes. Consistent with this view, diffusion tensor imaging metrics—including fractional anisotropy (FA) and mean diffusivity (MD)—differ significantly across vascular lesion types and healthy brain tissue. When FA values in normal-appearing white matter (NAWM) are weighted by white matter hyperintensity (WMH) volume, FA is significantly lower in individuals with hypertension, indicating microstructural disintegration of NAWM when accounting for WMH burden (79). As expected, the lowest values of MD and radial diffusivity (RD) are observed in NAWM, while axial diffusivity (AD) shows a similar pattern, with more pronounced differences between deep white matter hyperintensities and normal-appearing gray matter. Individuals with cardiovascular disease and hypertension consistently show lower NAWM FA, underscoring the impact of cerebrovascular dysfunction on white matter integrity.

Population-based evidence further implicates vascular and metabolic factors in brain microstructural alterations. In a rural Chinese cohort of older adults, Li et al. (80) reported that larger choroid plexus (CP) volume was associated with increased total and periventricular WMH burden, basal ganglia perivascular spaces, and higher peak width of skeletonized mean diffusivity and free water in periventricular and deep white matter. CP enlargement was also linked to higher body mass index, hypertension, diabetes, and cardiovascular disease, suggesting that CP volume may represent an early imaging marker of cerebral small vessel disease, although causal relationships cannot be inferred due to the cross-sectional design.

Interventional and metabolic studies further support the modulatory role of vascular health on brain structure. Lorenzon et al. (81) showed that individuals with diffuse or frontal-predominant cortical thinning but more favorable vascular profiles—characterized by lower blood pressure and reduced obesity—exhibited significantly less cortical thinning following intervention across global, Alzheimer’s disease–signature, and resilience-signature regions. Similarly, Soleymani et al. (82) reported associations between hemoglobin A1c levels and multiple brain metrics, including cortical thickness, diffusion measures, activity, and connectivity. Although findings were heterogeneous, hippocampal volume, WMH burden, and ventral attention network connectivity emerged as consistently affected, suggesting that glucose intolerance may influence brain structure and function even in the absence of overt metabolic disease.

Advanced diffusion analyses provide additional mechanistic insight. Using fixel-based analysis, Andica et al. (83) demonstrated a graded pattern of white matter damage across metabolic states, with early axonal loss in pre-metabolic syndrome and substantial axonal loss and fiber atrophy in overt metabolic syndrome, preferentially involving tracts supporting cognitive and motor function. In type 2 diabetes mellitus, Zebarth et al. (84) showed that increased white matter perivascular space volume mediated the effects of diabetes on multiple small vessel disease and diffusion markers, but only in individuals with coexisting hypertension, implicating perivascular fluid dynamics as a key pathway linking metabolic and vascular risk to cerebrovascular damage. Complementing these findings, Liu et al. (85) reported spatially selective alterations in commissural and association fibers in type 2 diabetes, with tract-specific diffusion metrics independently predicting cognitive performance and small vessel disease burden.

Finally, frailty represents an additional modifier of brain structure and microstructure. Gutiérrez-Zúñiga et al. (86) demonstrated that higher frailty index values were independently associated with lower cortical and thalamic volumes, particularly in orbitofrontal and temporal regions, as well as reduced integrity of selected association white matter bundles. Notably, only specific frailty components were directly related to brain measures, highlighting the need for frailty indices that more explicitly incorporate brain health–related metrics in aging research.

In conclusion, vascular risk factors, metabolic disease, and frailty can substantially influence diffusion MRI metrics and may explain a relevant proportion of the changes commonly attributed to healthy aging or early neurodegenerative processes. The frequent coexistence and incomplete control of these factors in clinical cohorts highlight the difficulty of disentangling age-related effects from early pathological alterations, underscoring the need for cautious interpretation of diffusion-based biomarkers in aging and prodromal disease stages.

### Biomarkers of neurodegeneration

Recent advances in diffusion MRI have enabled the identification of sensitive biomarkers capturing microstructural damage, extracellular fluid dysregulation, glymphatic dysfunction, and network-level degeneration across neurodegenerative diseases. In Alzheimer’s disease, advanced diffusion models—such as fixel-based analysis, free-water imaging, NODDI, and ALPS—demonstrate high sensitivity to early tau-driven white matter damage, glymphatic impairment, and cognitive decline, often preceding overt atrophy. Multimodal validation using PET, CSF, and plasma biomarkers consistently links diffusion-derived metrics to amyloid and tau pathology, metabolic dysfunction, astroglial injury, and small vessel disease.

Across Parkinson’s disease and synucleinopathies, diffusion MRI reveals convergent microstructural and glymphatic alterations related to motor laterality, cognition, sleep disorders, and disease progression. In multiple sclerosis and NMOSD, advanced diffusion and quantitative MRI outperform conventional tensor metrics in detecting myelin and axonal damage, lesion burden, and disability, supporting their role as sensitive markers of tissue injury. Overall, diffusion MRI biomarkers reflect a hierarchical progression from early microstructural and fluid-dynamic alterations to tract-specific degeneration, synaptic loss, and large-scale network disruption, positioning diffusion MRI as a systems-level framework for staging neurodegeneration.

Table 16 summarizes studies that reported clearly defined and robust reference standards, enabling a more reliable assessment of the prognostic performance of diffusion MRI biomarkers, whereas studies with less well-established or heterogeneous reference criteria are discussed qualitatively in the review (Table 17).

**Table 16:** Summary of studies using clear and robust reference standards.

Disease	Biomarker/method	Imaging/modality	Main findings	Clinical/biological relevance	Evidence
AD	Free-water correction (DL-based)	Diffusion MRI	Improved reliability and biological plausibility of diffusion metrics	Methodological robustness across acquisition schemes	(26)
AD	Fixel-based analysis (FBA)	Diffusion MRI + Tau-PET	Tau-related tract-specific WM degeneration not detected by DTI	Early tau-driven WM damage preceding atrophy	(145)
AD	FBA	Diffusion MRI	Resolves crossing fibers; fiber-specific metrics across aging	Methodological advantage over tensor models	(29)
AD	ALPS index	Diffusion MRI + Amyloid PET	Lower ALPS predicts amyloid accumulation, neurodegeneration, progression	Temporal role of glymphatic dysfunction	(96)
AD	PVSVF, Free Water, ALPS	Diffusion MRI + CSF + FDG-PET	↑ PVS & FW, ↓ ALPS associated with CSF Aβ42, hypometabolism, cognition	Multimodal validation of glymphatic impairment	(32)
AD	Superficial WM free water	Diffusion MRI + PET	Posterior-predominant abnormalities in preclinical AD	Very early microstructural marker	(87)
AD	Free water	Diffusion MRI + Plasma biomarkers	Distinguishes plasma Aβ42/40 + vs. − independent of PET	Blood-based stratification	(146)
AD	ALPS index	Diffusion MRI + Cognition	Associated with baseline and longitudinal cognitive decline	Predictive biomarker beyond plasma Aβ	(108)
AD	Free water	Diffusion MRI	Strongest association with decline across cognitive domains	Prognostic relevance	(147)
AD	NODDI	Diffusion MRI + PET	Higher sensitivity to medial temporal WM damage vs. DTI	Early AD WM alterations	(101)
AD + CSVD	ALPS, WMH, CP volume	Diffusion MRI + Amyloid PET	ALPS mediates amyloid/WMH effects on cognition	Glymphatic–vascular interaction	(94)
AD	AQP4 (CSF) + diffusion	Diffusion MRI + CSF	Glymphatic biomarkers vary with stage and damage	Molecular support for glymphatic failure	(105)
Aging / AD	NODDI, Free water	Diffusion MRI + Tau-PET	NODDI → brain health; FW → aging & early tau	Differential sensitivity	(100)
AD	PVS, WMH, GFAP	MRI + Plasma	Executive dysfunction mediated by GFAP & WM disease	Astroglial–vascular mechanism	(106)
AD	PSMD	Diffusion MRI	Predicts cognitive decline beyond amyloid/tau	Independent prognostic marker	(100)
AD	Multimodal MRI	MRI + PET	Improved GM change detection	Multimodal sensitivity	(27)
AD	α-synuclein (CSF)	CSF	Linked to tau pathology and neurodegeneration	Molecular prognostic marker	(115)
AD	Network-weighted degeneration	Diffusion MRI + Plasma + Post-mortem	Synaptic loss follows network topology	Systems-level degeneration	(148)
AD	FBA	Diffusion MRI	Distinct WM patterns in early vs. late-onset AD	Structural heterogeneity	(99)
PD	Bi-tensor DTI (FW-corrected)	Diffusion MRI	Asymmetric CC degeneration linked to motor laterality	Motor–nonmotor coupling	(47)
PD	NODDI	Diffusion MRI + Gait	WM changes correlate with gait (ON/OFF)	Early motor impairment	(120)
PD	VTA vs. SNc degeneration	Longitudinal MRI	Temporal dissociation, early VTA involvement	Pathophysiological staging	(115)
PD	ALPS + Free water	Diffusion MRI	↑ Thalamic FW, inverse ALPS–FW association	Glymphatic dysfunction	(117)
PD	ALPS index	Diffusion MRI	Reduced ALPS in PD, decline in sleep-disorder converters	Disease progression	(72)
PD	DTI-ALPS	Diffusion MRI	Identifies PDD converters with high accuracy	Dementia prediction	(110)
PD	CSF motion	Multi-shell diffusion MRI	Reduced suprasellar CSF motion	Glymphatic flow alteration	(111)
iRBD	ALPS index	Diffusion MRI	Correlates with cognition, olfaction, limbic volumes	Prodromal marker	(149)
PD	DKI connectivity	Diffusion MRI	Network disorganization	Network-level degeneration	(119)
MS	ALPS index	Diffusion MRI	Lower ALPS linked to lesions, atrophy, NAWM damage	Glymphatic involvement	(131)
MS	μFA	Diffusion MRI	More sensitive than FA to lesions MS	Microstructural sensitivity	(150)

**Table 17:** Early vs. Late Diffusion MRI Biomarkers of Neurodegeneration.

Domain	Early biomarkers (preclinical/prodromal)	Late biomarkers (established neurodegeneration)
White-matter microstructure (DTI)	↓ FA, ↑ MD/RD reflecting subtle demyelination and early axonal disorganization (9–11, 151, 152)	Marked FA reduction and diffuse MD/RD increase reflecting advanced axonal loss and tract degeneration (28, 29, 120)
Gray-matter microstructure (DKI)	↓ MK and RK indicating early dendritic and synaptic simplification preceding atrophy (8, 30, 113)	Severe kurtosis reduction associated with neuronal loss and cortical thinning (30, 47)
Neurite integrity (NODDI)	↓ NDI, ↑ ISOVF, altered ODI reflecting early neuritic loss and extracellular expansion (100, 101, 125, 126)	Profound NDI reduction with widespread neurite collapse and network disintegration (30, 134)
Fiber-specific degeneration (FBA)	Focal ↓ FD and/or FC in vulnerable tracts predicting clinical progression (28, 77, 99)	Extensive FD, FC, and FDC loss reflecting irreversible tract atrophy (29, 120)
Extracellular fluid dynamics (FWI)	↑ FW indicating early neuroinflammation, BBB dysfunction, and impaired clearance (96, 113, 114, 130)	Marked FW expansion associated with tissue rarefaction and advanced degeneration (47, 117, 153)
Perivascular/glymphatic function (ALPS, PVS)	↓ ALPS, mild PVS enlargement reflecting early glymphatic inefficiency (23, 32, 96, 116)	Severe ALPS reduction and prominent PVS enlargement indicating glymphatic failure (33, 40, 94, 110, 154)
Network organization	Reduced structure–function coupling and selective hub vulnerability (99–101)	Global network disconnection and loss of integration (29, 120)
Clinical correlates	Subtle cognitive changes, subjective cognitive decline, prodromal motor/non-motor symptoms (100, 113, 114)	Overt cognitive impairment, dementia, severe motor or functional disability (110, 117, 154)

## Pathophysiology of brain aging and neurodegeneration

### Vasomotor pump failure

The collapse of the vasomotor pump reflects a hierarchical failure across three tightly coupled levels rather than an isolated defect (35). At the upstream level, degeneration of the locus coeruleus undermines the noradrenergic infralow rhythms that normally synchronize vascular tone, astrocytic activity, and sleep-dependent clearance. This loss of temporal precision precedes overt structural degeneration and sets the stage for downstream vulnerability.

At the intermediate level, cerebrovascular stiffening prevents rhythmic neuromodulatory input from being effectively converted into mechanical vasomotion. Structural and phenotypic remodeling of the vascular wall narrows the dynamic range of diameter oscillations and disrupts the multiscale integration of infralow, cardiac, and respiratory pressure components required for convective transport.

At the downstream level, disruption of the astro-glial perivascular interface imposes a final hydraulic bottleneck. Loss of astrocytic polarity and impaired dynamic regulation of perivascular geometry markedly increase resistance to CSF–ISF exchange, rendering clearance ineffective even when upstream signaling or mechanical components are partially preserved.

Together, these three failures form a self-reinforcing system in which impaired neuromodulatory drive, mechanical decoupling, and interface breakdown converge to shift the brain from an active, pump-driven clearance regime to a diffusion-limited state. This integrated collapse provides a mechanistic framework linking aging, vascular disease, sleep disruption, and neurodegeneration (Table 18).

**Table 18:** Multilevel mechanisms underlying collapse of the vasomotor pump.

Level of failure	Primary driver	Key cellular/molecular alterations	Mechanistic consequence	Functional impact on clearance
1 Noradrenergic drive (LC degeneration)	Aging, oxidative stress, mitochondrial dysfunction, systemic inflammation, BBB breakdown	Loss of LC neurons projecting to forebrain vasculature and astro-glial territories; altered HCN channels, reduced T-type Ca^2+^ currents, disrupted GABAergic modulation; reduced α1- and β-adrenergic signaling	Loss of precision and coherence of infralow noradrenergic oscillations; decoupling of LC output from cortical slow waves and astrocytic Ca^2+^ rhythms	Reduced vasomotor gain and temporal coordination; impaired entrainment of vascular and glial components; early failure of clearance timing
2 Mechanical transduction (vascular stiffening)	Aging, hypertension, metabolic disorders, oxidative stress, AGEs	Elastin loss, collagen cross-linking, internal elastic lamina fragmentation; VSMC phenotypic switch (contractile → synthetic); impaired Ca^2+^ dynamics; microvascular rarefaction and pericyte loss	Attenuation of diameter oscillations; loss of frequency-layered coupling between vasomotion, cardiac and respiratory pulsatility; collapse of multiscale pressure gradients	Failure to generate convective perivascular flow; transport becomes diffusion-limited and inefficient for clearing proteins and metabolites
3 Perivascular interface (astro-glial disruption)	Astrocytic reactivity, microglial activation, immune infiltration	AQP4 depolarization from astrocytic end-feet; disruption of dystrophin–dystroglycan complex; reduced IP3 receptor expression and Ca^2+^ signaling; basement-membrane remodeling	Increased hydraulic resistance at the CSF–ISF interface; impaired dynamic adjustment of perivascular geometry	Marked reduction of CSF influx and ISF exchange (>50%); solute retention and stagnation despite preserved upstream inputs
Integrated outcome	Convergent failure across levels	LC degeneration + vascular stiffening + astro-glial depolarization	Transition from active convective pump to diffusion-limited architecture	Ineffective clearance of amyloid-β, tau, α-synuclein, inflammatory mediators

Within the Conformation–Propagation–Microenvironment framework, neurodegeneration emerges from the interaction between protein misfolding, network topology, and failure of the brain microenvironment. Synapses act as the critical interface where activity-dependent propagation of misfolded proteins converges with metabolic and inflammatory stress, rendering highly connected networks selectively vulnerable.

Degeneration of the locus coeruleus disrupts noradrenergic infralow rhythms that normally coordinate neuronal activity, vasomotion, astrocytic signaling, and sleep-dependent clearance. This upstream failure prevents effective coupling between neuromodulatory drive and vascular mechanics, a process further amplified by age- and disease-related vascular stiffening.

**Figure 3:**
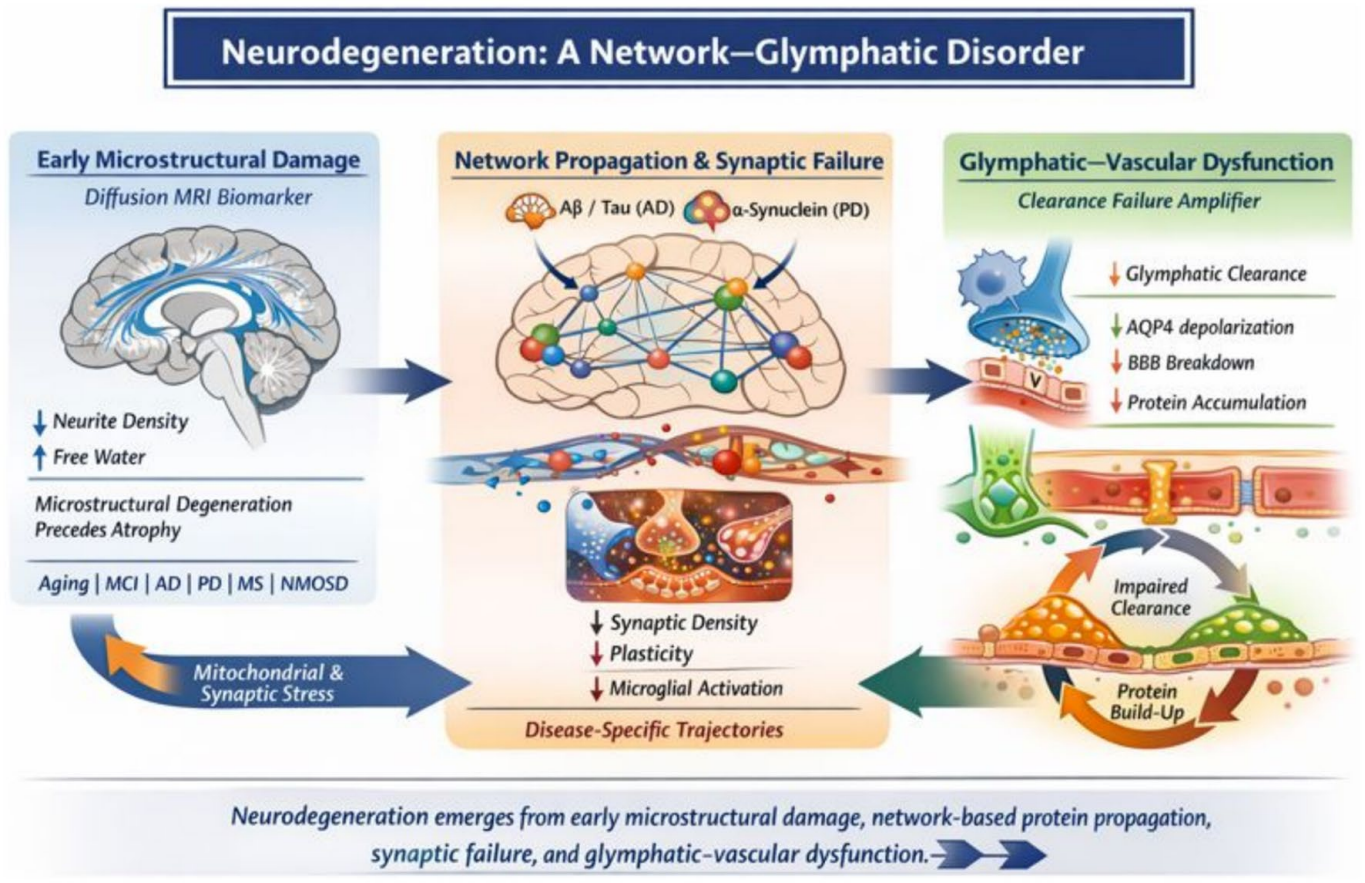
The schematic illustrates a unified model of neurodegeneration, starting from early microstructural damage detectable by diffusion MRI biomarkers (reduced neurite density and increased free water), preceding overt atrophy. Progressive mitochondrial and synaptic stress promotes network-based propagation of pathological proteins (Aβ and tau in Alzheimer’s disease, *α*-synuclein in Parkinson’s disease), leading to synaptic loss, reduced plasticity, and microglial activation. Concurrent glymphatic–vascular dysfunction, characterized by impaired glymphatic clearance, AQP4 depolarization, blood–brain barrier disruption, and protein accumulation, amplifies disease progression along disease-specific trajectories.

At the downstream level, disruption of the astro-glial perivascular interface imposes a hydraulic bottleneck that renders glymphatic exchange ineffective, even when upstream components are partially preserved. The combined failure across molecular, network, neuromodulatory, vascular, and glial levels shifts the brain from an active, pump-driven clearance system to a diffusion-limited state, providing a unifying mechanistic substrate for neurodegeneration across disorders. Table 19 proposes an integrated generative model based on the CPM framework and vasomotor pump failure Figure 3 illustrates selected pathophysiological mechanisms discussed in the text.

**Table 19:** Conceptual integrated model of neurodegenerative pathophysiology based on CPM and vasomotor pump dysfunction.

Level	Core process	Key mechanisms	Network/clearance consequence
Molecular	Protein misfolding and propagation	Soluble Aβ and tau with prion-like, activity-dependent spread at synapses	Initiation of synaptotoxic cascades and local metabolic stress
Synaptic/Network	Selective network vulnerability	Propagation constrained by connectivity; early involvement of DMN and medial temporal hubs	Accelerated trans-synaptic spread and early network destabilization
Neuromodulatory	LC degeneration	Loss of noradrenergic infralow rhythmicity regulating excitability, sleep, and clearance	Reduced synchronization of neuronal, vascular, and glial activity
Vascular	Impaired vasomotion	Vascular stiffening limits translation of neuromodulatory signals into diameter oscillations	Failure of convective perivascular transport
Astro-glial interface	Perivascular dysfunction	AQP4 depolarization and loss of astrocytic polarity increase CSF–ISF resistance	Ineffective glymphatic exchange despite preserved upstream inputs
System-level outcome	Clearance collapse	Transition from pump-driven to diffusion-limited regime	Accumulation of toxic proteins, sustained inflammation, cognitive decline

## Discussion

This review adopts the neuro–glio–vascular unit (NGVU) as a unifying framework for neurodegeneration, integrating neuronal, glial, vascular, and perivascular components into a single functional system governing neurovascular coupling, metabolic support, immune surveillance, and waste clearance (87, 88). Within this perspective, diffusion MRI metrics reflect the integrated brain microenvironment rather than isolated tissue damage. Alterations in neurite density, diffusion kurtosis, free-water fraction, perivascular diffusivity, and the ALPS index capture complementary aspects of NGVU dysfunction, including astrocytic AQP4 dysregulation, blood–brain barrier instability, impaired vascular pulsatility, and reduced glymphatic clearance. This framework reconciles heterogeneous diffusion findings across aging and neurodegenerative diseases, providing a common mechanistic substrate for convergent microstructural and fluid-dynamic abnormalities in Alzheimer’s disease, Parkinson’s disease, multiple sclerosis, and neuromyelitis optica spectrum disorder.

Historically, Alzheimer’s disease could be definitively diagnosed only by post-mortem neuropathological examination demonstrating *β*-amyloid plaques and neurofibrillary tangles. Despite major advances in *in vivo* biomarkers, clinical diagnosis remains substantially delayed, with a typical 20–50-month interval between symptom onset and formal diagnosis. These delays have become increasingly consequential with the introduction of disease-modifying anti-amyloid therapies, which require biological confirmation of pathology rather than purely clinical criteria. Current appropriate-use recommendations mandate objective evidence of β-amyloid deposition—obtained through amyloid PET, cerebrospinal fluid biomarkers, or validated blood-based markers such as plasma p-tau217—and restrict treatment to early disease stages, accompanied by MRI-based safety monitoring. Biomarker confirmation is therefore essential for diagnosis, therapeutic eligibility, and risk stratification.

To reduce diagnostic delays, scalable biomarker-driven diagnostic pathways have been proposed, integrating cognitive screening with blood-based biomarkers in primary care, followed by targeted confirmatory testing. These strategies are grounded in the A–T–N framework, which classifies Alzheimer’s disease according to *β*-amyloid deposition (A), tau pathology (T), and neurodegeneration (N). Amyloid biomarkers typically become abnormal early, often decades before symptom onset; tau biomarkers show the strongest association with synaptic dysfunction and cognitive decline; and neurodegeneration markers—including structural MRI atrophy, FDG-PET hypometabolism, and neurofilament light in cerebrospinal fluid and blood—reflect downstream neuronal injury and correlate most closely with clinical severity, albeit with limited disease specificity. Importantly, the A–T–N framework accommodates biological heterogeneity and supports a dynamic, multistage view of the disease.

Diffusion MRI functions as a systems-level imaging modality capable of capturing NGVU breakdown across neurodegenerative diseases. When integrated within a biomarker-first, A–T–N–aligned framework and combined with molecular biomarkers and standardized longitudinal strategies, diffusion-derived metrics hold substantial potential for early disease stratification, monitoring, and therapeutic targeting.

Among diffusion-based approaches, conventional DTI metrics and free-water imaging have been reported to show associations with histopathological features of microstructural tissue alteration in selected contexts; however, these relationships remain indirect and model-dependent. Techniques such as ALPS and perivascular space–related measures should be interpreted with particular caution, as they are considered indirect surrogates that primarily reflect fluid-related and compartmental dynamics rather than specific, well-validated anatomo-pathological correlate.

As discussed by Murray (89), the term *neuropathology* should be reserved for tissue-based investigations of the human brain, clearly distinguishing these studies from biofluid biomarkers that act as peripheral indicators of underlying disease processes. Advances in high-throughput digital whole-slide imaging now enable computerized, quantitative analysis of tissue morphology and structural disease patterns, a field increasingly referred to as *pathomics*.

This transformative evolution in neuropathology integrates traditional descriptive pathology with advanced multi-omics approaches and machine learning techniques, redefining human brain research. By enabling comprehensive, high-dimensional analyses of postmortem brain tissue, these methodologies are expected to accelerate the identification of disease biomarkers, therapeutic targets, and complex pathological patterns underlying neurodegenerative disorder.

At the same time, it is important to acknowledge the risk of technology enthusiasm and to emphasize that advances in diffusion MRI and artificial intelligence should be critically evaluated in relation to patient-centered outcomes, real-world clinical feasibility, and cost-effectiveness, rather than technical performance alone.

## Limitations

The available evidence is largely observational and heterogeneous, limiting causal inference. Methodological variability across studies—including scanner vendor, hardware performance, acquisition protocols, and processing pipelines—could not be controlled and likely contributes substantially to inter-study variability in diffusion-derived measures.

Diffusion MRI provides indirect proxies of tissue microstructure and cannot be directly equated with histology, as water diffusion is influenced by physiological and metabolic factors absent post-mortem. As a result, biological specificity remains limited, particularly when diffusion metrics are interpreted as direct surrogates of cellular or subcellular processes. Model-dependent assumptions, incomplete histological validation, and inadequate control of comorbid conditions further constrain mechanistic interpretation and limit the specificity of conclusions of this review. Diffusion-derived metrics should therefore be regarded as relative *in vivo* biomarkers, particularly suited for longitudinal monitoring.

Glymphatic-related markers, including the ALPS index and perivascular space metrics, are influenced by multiple physiological and clinical factors such as sleep, circadian phase, blood pressure, vascular comorbidity, hydration status, and medication use. Because these factors are not consistently controlled, reported associations with aging or neurodegeneration may be affected by residual confounding.

An additional limitation concerns the statistical robustness of connectomic findings. Many studies do not consistently correct for multiple comparisons across all tested metrics and nodes, increasing the risk of false-positive network results. While this review summarizes such findings, their robustness cannot be guaranteed, and independent replication in larger, well-powered cohorts remains necessary.

Clinical translation is further limited by feasibility, reproducibility, and standardization challenges. Advanced diffusion models and AI-based approaches require demanding acquisitions, are sensitive to scanner variability, and may not generalize across sites, with additional risks of overfitting and limited external validation. The clinical impact of AI-based diffusion MRI methods remains under evaluation, and the present review may over-represent early and potentially optimistic studies, while longer-term evidence from large-scale, real-world clinical implementation is still limited.

Technical factors—including signal-to-noise ratio, spatial resolution, motion sensitivity, and partial-volume effects—together with the predominantly retrospective nature of the literature, introduce additional bias and highlight the need for prospective, biomarker-driven longitudinal studies. Overall, diffusion MRI–based biomarkers should currently be considered complementary rather than stand-alone tools for clinical decision-making.

In addition, this review did not systematically evaluate health-economic data, which represents an important area for future research to inform cost-effectiveness and clinical implementation of advanced diffusion MRI biomarkers. Publication bias and selection bias are likely. This review summarizes the available evidence but cannot guarantee the robustness of the reported findings; independent replication in larger and well-characterized cohorts is therefore required.

## Conclusion

In summary, the evidence reviewed supports diffusion MRI as a complementary, systems-level imaging approach capable of capturing neuro–glio–vascular unit alterations across neurodegenerative diseases when interpreted within a multimodal biomarker framework. In current clinical practice, diffusion-derived metrics should not be used as stand-alone diagnostic tools but may contribute to early stratification, longitudinal monitoring, and treatment evaluation when integrated with molecular biomarkers and standardized imaging protocols.

From a research perspective, future studies should prioritize prospective, multi-center designs with harmonized acquisition and processing pipelines, robust reference standards, careful control of confounding factors, and explicit correction for multiple comparisons. Particular emphasis should be placed on external validation, clinical feasibility, and cost-effectiveness to facilitate translation from experimental diffusion biomarkers to clinically meaningful decision support.

### Review methods

Narrative (non-systematic) review. Searches were performed in PubMed, first without year restrictions, then limited to the last 5 years, and subsequently to the last 1 year, using the following terms: NVU, DWI, dMRI, techniques, DMI, neurodegeneration, aging, MCI, Alzheimer’s disease, Parkinson’s disease, MS, NMOSD, brain connectivity, microstructure, dementia, glymphatic system, DTI, sleep, PVS, voxelwise, TBSS, free water, tractography, DTI-ALPS, choroid plexus, thalamus, hippocampus, myelin, artificial intelligence, circadian rhythm, hypertension, CSVD, medications, CSF, hydration status, vascular risk factors, metabolic disease, frailty, connectomics, and exploratory analyses.

Inclusion criteria were studies addressing etiopathogenesis, reporting experimental data, and/or review articles based on large study samples or large patient cohorts.

## Glossary

AD: Alzheimer’s Disease
AI: Artificial Intelligence
ALPS: Analysis along the Perivascular Space
AQP4: Aquaporin-4
AxD: Axial Diffusivity
BBB: Blood–Brain Barrier
CC: Corpus Callosum
CP: Choroid Plexus
CSF: Cerebrospinal Fluid
CSVD: Cerebral Small Vessel Disease
CSTC: Cortico–Striato–Thalamo–Cortical
dMRI: Diffusion Magnetic Resonance Imaging
DKI: Diffusion Kurtosis Imaging
DMI: Diffusion Microstructure Imaging
DMN: Default Mode Network
DTI: Diffusion Tensor Imaging
DTI-ALPS: Diffusion Tensor Imaging–Analysis along the Perivascular Space
DWI: Diffusion-Weighted Imaging
FA: Fractional Anisotropy
FBA: Fixel-Based Analysis
FC: Fiber Cross-section
FD: Fiber Density
FDC: Fiber Density and Cross-section
FDG-PET: Fluorodeoxyglucose Positron Emission Tomography
FW: Free Water
GFAP: Glial Fibrillary Acidic Protein
GM: Gray Matter
HC: Healthy Controls
ISF: Interstitial Fluid
ISOVF: Isotropic Volume Fraction
LC: Locus Coeruleus
MD: Mean Diffusivity
MCI: Mild Cognitive Impairment
MRI: Magnetic Resonance Imaging
MS: Multiple Sclerosis
MT: Magnetization Transfer
NAWM: Normal-Appearing White Matter
NMOSD: Neuromyelitis Optica Spectrum Disorder
NODDI: Neurite Orientation Dispersion and Density Imaging
ODI: Orientation Dispersion Index
PD: Parkinson’s Disease
PDD: Parkinson’s Disease Dementia
PET: Positron Emission Tomography
PSV: Perivascular Space Volume
PVS: Perivascular Spaces
RD: Radial Diffusivity
RBD: Rapid Eye Movement Sleep Behavior Disorder
ROI: Region of Interest
SNR: Signal-to-Noise Ratio
SNpc: Substantia Nigra pars compacta
TBSS: Tract-Based Spatial Statistics
VBM: Voxel-Based Morphometry
WM: White Matter
WMH: White Matter Hyperintensities
